# Consistent multiscale modelling of movement and habitat selection

**DOI:** 10.1007/s00285-026-02370-w

**Published:** 2026-05-20

**Authors:** Paul G. Blackwell

**Affiliations:** School of Mathematical and Physical Sciences, University of Sheffield, Sheffield, UK

**Keywords:** Diffusion, Markov chain Monte Carlo, Piecewise deterministic Markov process, Step selection, Velocity-jump process

## Abstract

In spatial ecology, the concept of resource selection expresses the idea that for many animals, the distribution of an individual’s location is not uniform over the region available to them; instead, they spend time preferentially in some locations compared to others, in a way that can often be related to spatial covariates. The related concept of step selection describes the variation in an individual’s tendency to move to particular locations in the short term, taking into account both spatial covariates and the constraints of their process of movement. Consistent modelling of resource selection and step selection is necessary to understand animals’ distribution in space and to interpret movement, telemetry, and spatial survey data in a meaningful way. In this paper, I take advantage of recent developments in stochastic processes and statistical algorithms to develop a range of new stochastic models in which both the dynamics and the long-term behaviour are tractable and described parametrically, and which are flexible enough to represent a wide range of patterns of movement and space use encountered in reality. I extend the mathematical analogy between movement modelling and Markov chain Monte Carlo algorithms, first proposed by Michelot, Blackwell & Matthiopoulos (2019; *Ecology* **100**, e02452), to a wide range of continuous-time stochastic processes, including both diffusion processes and velocity-jump models, that in different ways are motivated by the simple discrete-time step-and-turn models widely used in practice. Particular cases include a diffusion process where the dynamics are defined in terms of speed and direction of movement, and a velocity-jump process in *d* dimensions, generalizing the ‘bouncy particle sampler’ used in Bayesian inference, in which the distribution of velocity after a so-called ‘bounce’ event has support over a region which itself has dimension *d*. I also show how this mathematical approach can be extended to models incorporating distinct behavioural states and to higher dimensional models representing the joint movement of interacting individuals.

## Introduction

The aim of this paper is to explore the mathematical relationship between models of the movement of animals and models of their space use, i.e. where they spend their time, expressed as both short-term and long-term selection based on resources or other habitat features. Here, and throughout the paper, ‘selection’ refers to animals preferentially spending time in some locations rather than others; while presumably the movement behaviour is the result of evolutionary pressures, selection in the evolutionary sense is beyond the scope of this work.

An animal’s long-term space use, when it is well defined, is expressed as its utilisation distribution. For the purposes of this paper, I will assume that this is fully described by a density function which I will denote $\pi (\textbf{x})$ at a location $\textbf{x}$ in *d*-dimensional space, and is not time-dependent. The density $\pi (\textbf{x})$ defines the probability of finding the animal at a given location at an arbitrary instant in time.

Selection in the sense used here is essentially about the relationship between $\pi (\textbf{x})$ and spatially varying covariates $c_k(\textbf{x})$. These covariates are not necessarily wholly local; for example a particular $c_k(\textbf{x})$ might measure prey density or temperature at $\textbf{x}$, but could for example measure distance of $\textbf{x}$ from a nest site or from the nearest water source, or the proportion of tree cover over a disc of a specified radius centred at $\textbf{x}$. The $c_k(\textbf{x})$ are assumed to be constant over time, like $\pi (\textbf{x})$ itself.

As an illustration, a linear-exponential form is often used:

1 $$\begin{aligned} \pi (\textbf{x})&\propto \exp \left( \sum _{k=1}^K\beta _kc_k(\textbf{x})\right) \end{aligned}$$

2 $$\begin{aligned}&= \exp ({L}(\textbf{x})) \end{aligned}$$

say, where the logarithm of the utilisation density is modelled as a linear combination of the covariates (the ‘linear predictor’); see e.g. Boyce and McDonald (1999). The coefficients $\beta _k$ are termed selection parameters. Selection of this kind is often termed habitat selection or resource selection, and is typically used in conjunction with spatial survey data which can be thought of as being sampled directly from $\pi (\textbf{x})$.

In contrast, to think about an animal’s short-term ‘preferences’, typically a conditional model is used, which aims to model the conditional distribution of the animal’s location $\textbf{x}(t+\delta t)$ at time $t+\delta t$ in terms of its location $\textbf{x}(t)$ at time *t*, or more generally of a set $\mathcal {H}_t$ of earlier locations including $\textbf{x}(t)$, and of local values of covariates. Often this is referred to as step selection, where ‘step’ is used conventionally to refer to the movement between one time and another (e.g. Avgar et al. 2016). Here I will adopt that terminology, as a useful distinction from resource selection, meaning the modelling of the distribution of $\textbf{x}$ unconditionally; but note that sometimes the term step selection is used more narrowly (e.g. Thurfjell et al. 2014). In practice, the times *t* and $t+\delta t$ are generally the times of successive observations made using some form of telemetry, e.g. from GPS tags, and so a step refers to the change in location between one observation and the next. Almost all existing approaches to step selection express the conditional distribution $p(\textbf{x}(t+\delta t)|\mathcal {H}_t)$ is a product form, generally referred to as using weighted distributions (Johnson et al. 2008b) or as a separable model (Avgar et al. 2016):

$$ p(\textbf{x}(t+\delta t)|\mathcal {H}_t,\textbf{c}(\textbf{x}(t+\delta t))) \propto w(\textbf{c}(\textbf{x}(t+\delta t))) \phi (\textbf{x}(t+\delta t)|\mathcal {H}_t), $$

where $\textbf{c}= (c_1,\ldots ,c_K)$, $w(\textbf{c})$ is a selection function incorporating the effects of spatial covariates and $\phi (\textbf{x}|\mathcal {H})$ is a movement kernel. Johnson et al. (2013) formulate their models rather differently, as spatio-temporal point processes, allowing additional techniques to be used in implementation, but the same considerations below still apply.

Again, it is common to use a parametric form for both functions $w(\textbf{c})$ and $\phi (\textbf{x}|\mathcal {H})$ defining the step selection; see e.g. Thurfjell et al. (2014). A simple example would be to allow the movement kernel to depend on distance moved from the previous location, so that $\mathcal {H}_t$ is just $\{\textbf{x}(t)\}$ and

$$ \phi (\textbf{x}(t+\delta t)|\textbf{x}(t)) \propto f(||\textbf{x}(t),\textbf{x}(t+\delta t)||), $$

where $||\cdot ,\cdot ||$ represents the distance between locations, and to write $w(\textbf{c})$ in linear-exponential form

3 $$\begin{aligned} w(\textbf{c}(\textbf{x})) = \exp \left( \sum _{k=1}^K\beta _k^* c_k(\textbf{x})\right) . \end{aligned}$$

Then

4 $$\begin{aligned} p(\textbf{x}(t+\delta t)|\textbf{x}(t)) \propto f(||\textbf{x}(t),\textbf{x}(t+\delta t)||) \cdot \exp \left( \sum _{k=1}^K\beta _k^* c_k(\textbf{x}(t+\delta t))\right) . \end{aligned}$$

That is, the probability distribution for the new location $\textbf{x}(t+\delta t)$ is proportional to an exponential function of the covariates, evaluated at that new location, and also proportional to some function of its distance from the previous location $\textbf{x}(t)$. The coefficients $\beta _k^*$ are interpreted as selection parameters. The notation used here reflects the obvious parallel between Eqs. (1) and (3), without assuming *a priori* that the parameters have identical interpretations; in fact they do not, which is a key motivation for the work presented here. More typical models in practice include two previous locations in $\mathcal {H}$ and use a movement kernel that involves not only the distance moved but also the turning angle between successive ‘steps’ (Thurfjell et al. 2014), but this does not avoid any of the problems addressed here, and adds further issues of interpretation. For simplicity I will not describe that refinement in detail, but models that do include velocity, and hence some concept of turning angle, are included in later sections. For a more comprehensive coverage of existing approaches to resource selection, see Matthiopoulos et al. (2023) and, specifically in the context of movement and telemetry, Hooten et al. (2017).

An obvious problem with this sort of formulation of step selection, in discrete time, is that it ignores locations at intervening times, even though in most cases $\delta t$ is arbitrary as far as the animal’s movement is concerned. (Some exceptions are discussed briefly in §8.) This can be addressed by formulating models in continuous time instead (see Sects. 3 and 4), and naturally becomes less problematic as $\delta t$ decreases.

A more fundamental issue with this approach—raised by Moorcroft and Barnett (2008), and addressed in very specific cases by Barnett and Moorcroft (2008), Mich-elot, Blackwell and Matthiopoulos (2019a), Michelot et al. (2020), Michelot et al. (2019b)—is that the selection function $w(\textbf{c}(\textbf{x}))$ is not equivalent to the utilisation distribution $\pi (\textbf{x})$. That is, the long-term distribution of an individual that moves around according to Eq. (4) is not the distribution defined by $\pi (\textbf{x}) \propto w(\textbf{c}(\textbf{x}))$; a separable model with selection function $w(\textbf{c}(\textbf{x}))$ does not give rise to a utilisation distribution $\pi (\textbf{x}) \propto w(\textbf{c}(\textbf{x}))$. In particular, if the linear-exponential forms in Eqs. (1) and (3) are used for the utilisation distribution and the selection function respectively, then the step-selection parameters $\beta _1^*,\ldots ,\beta _K^*$ are not the same as $\beta _1,\ldots ,\beta _K$, despite the apparent similarity of the models. The long-term distribution of an individual that moves around according to Eq. (4) is not the distribution that would be given by Eq. (1) with each $\beta _k=\beta _k^*$. In fact, following Eq. (4) does not typically lead to *any* utilisation distribution of the form in Eq. (1). In general, the selection function in a step-selection model does not have any direct interpretation in terms of the utilisation distribution, with some very specific exceptions (Moorcroft and Barnett 2008; Barnett and Moorcroft 2008) discussed below. These are two genuinely distinct representations of selection. This is not a problem of approximation; it is not resolved by taking $\delta t$ to be small. Nor is it purely an abstract problem. If an animal does have a stationary distribution $\pi (\textbf{x})$—whether it can be easily described mathematically or not—then its actual movement process must be consistent with that. There is no guarantee that the movement can be modelled using a simple form, but assuming a mathematical or parametric form that is inconsistent will inevitably lead to contradictory interpretation. A practical consequence arises in situations where it is possible to get both survey data and movement data on the same animals. It should be feasible to use such data jointly to learn about where an animal spends its time, but that is impossible using two representations of the process that are inconsistent. Blackwell and Matthiopoulos (2024) discuss this situation in detail, and show how such joint inference can be achieved in practice in a coherent way, as outlined here in §7. They use the Langevin model described in Sect. 3.1; a key motivation for the work here is to develop a much wider range of models that can be used in a similar way.

A further fundamental issue is that, even thinking only about step selection, the selection function $w(\textbf{c}(\textbf{x}))$, or selection parameters $\beta _k^*$, defined by locations on any one particular choice of timescale $\delta t$—in practice typically by regular observations—are not necessarily related in any systematic way to those given by any other timescale, even though, again, if they are meaningful at all then they ought to be consistent across timescales. Fundamentally this is because a separable model does not generally satisfy any Chapman-Kolmogorov-type criterion requiring that conditional distributions are consistently defined regardless of which time points are considered. For example, consider a separable model where, for simplicity, $\mathcal {H}_t = \{\textbf{x}(t)\}$. For times $t_0<t_1<t_2$, the distribution $p(\textbf{x}(t_2)|\textbf{x}(t_0))$ can be defined in two ways. Directly, we have

$$ p(\textbf{x}(t_2)|\textbf{x}(t_0)) \propto w(\textbf{c}(\textbf{x}(t_2))) \phi (\textbf{x}(t_2)|\textbf{x}(t_0)) $$

or if $t_1$ is considered,

$$\begin{aligned} p(\textbf{x}(t_2)|\textbf{x}(t_0))&= \int _{\textbf{x}(t_1)} p(\textbf{x}(t_2), \textbf{x}(t_1)|\textbf{x}(t_0) {{\text {d}}\textbf{x}}(t_1)\\&= \int _{\textbf{x}(t_1)} p(\textbf{x}(t_2)|\textbf{x}(t_1)) p(\textbf{x}(t_1)|\textbf{x}(t_0)) {{\text {d}}\textbf{x}}(t_1)\\&= \int _{\textbf{x}(t_1)} \frac{w(\textbf{c}(\textbf{x}(t_2)))\phi (\textbf{x}(t_2)|\textbf{x}(t_1))}{\mathcal {K}_2} \frac{w(\textbf{c}(\textbf{x}(t_1)))\phi (\textbf{x}(t_1)|\textbf{x}(t_0))}{\mathcal {K}_1} {{\text {d}}\textbf{x}}(t_1)\\&= w(\textbf{c}(\textbf{x}(t_2)))\int _{\textbf{x}(t_1)} \frac{w(\textbf{c}(\textbf{x}(t_1)))\phi (\textbf{x}(t_2)|\textbf{x}(t_1))\phi (\textbf{x}(t_1)|\textbf{x}(t_0))}{\mathcal {K}_1\mathcal {K}_2} {{\text {d}}\textbf{x}}(t_1) \end{aligned}$$

where each $\mathcal {K}$ is a normalising constant. Clearly in general these are not equivalent. This lack of consistency remains even when the movement kernel is formulated in a coherent way in continuous time, for example the diffusion processes used by Johnson et al. (2008b, §4.4). In that case, consistency criteria such as

$$ p(\textbf{x}(t_2)|\textbf{x}(t_0)) = \int _{\textbf{x}(t_1)} p(\textbf{x}(t_2)|\textbf{x}(t_1)) p(\textbf{x}(t_1)|\textbf{x}(t_0)) {{\text {d}}\textbf{x}}(t_1) $$

would be satisfied by the movement in the absence of selection (i.e. with $w(\textbf{c})$ constant) but not once selection was introduced through a separable model. Potentially, if all the values of $\delta t$ under consideration are sufficiently small, then this problem is straightforward to resolve, though not automatic. But in practice, there are many cases when step selection is based on data that do not meet this criterion; and even when they do, generally the issue is ignored. Again, this has practical consequences. Step selection from data at irregular times, i.e. when $\delta t$ varies, is not in general well-defined; and comparison between datasets collected on different timescales, when $\delta t$ differs between datasets even if not within them, is likely to be misleading. A partial exception is the time-varying step selection of Munden et al. (2021), which models the location and timing of turning points in a fully observed trajectory consisting of straight line segments, as in Sect. 4. However, even though the timescale there is a naturally emergent one, selection on that timescale does not map directly onto selection over other timescales.

The first of these fundamental issues can, of course, be addressed by ‘brute force’ to some extent. Any model of movement and selection *may* define a utilisation distribution, which can then be explored by approximation or simulation. For example, Avgar et al. (2016) obtain utilisation distributions by simulation after fitting a separable model, in which spatial covariates are included in the movement kernel as well as the selection function, and Potts et al. (2022) embed a simulation approach within an iterative process of refining separable models on the basis of the utilisation distributions obtained. Potts and Borger (2023) use a combination of simulation and approximation by partial differential equations to obtain utilisation distributions for separable models that incorporate additional complex features of memory and interactions between individuals. Signer et al. (2024) also provides detailed tools for stochastic simulation to obtain utilisation distributions. However, these approaches do not obviate the need for consistency over different timescales, and have several drawbacks. Firstly, such a model may in fact not define a utilisation distribution, if it is not a stationary process, and it is not always easy to see whether an arbitrary movement process is stationary. Blackwell and Matthiopoulos (2024) discuss a number of ways in which a movement model may be stationary by construction, expressed in biological terms. Secondly, because of the timescale inconsistencies described above, the utilisation distribution obtained in this way from a separable model will depend on the timescale used. For example, Avgar et al. (2016) “explicitly acknowledge that [their] inference is scale dependent” (their p. 622). Thirdly, the utilisation distribution generated in such a way will be hard to relate in a simple way to covariate information, and so will be hard to interpret and give limited insight. It is not obvious, from the point of view of parsimonious modelling, that it is sensible to apply a very simple model of selection at one arbitrary timescale if it leads to an uninterpretable one at other timescales or for long-term patterns. Finally, from a statistical point of view, this approach will generally give rise to a utilisation distribution that is not tractable, making it harder to combine information of different types as discussed above.

The solution to the fundamental problems of the inconsistencies of utilisation distributions and step-selection models—of stationary distributions and conditional distributions on various timescales—is to ensure that they are constructed in a consistent way. The idea of the current paper is to develop and explore plausible, tractable movement models *and* utilisation distributions that are consistent by construction. In part, that can be achieved by turning the problem round to ask: given a tractable utilisation distribution, defined parametrically in terms of known covariates, how can we construct plausible, tractable movement models consistent with it? This will broaden the range of movement models that can be used in this way, allowing for much more flexibility and realism. In some cases, incorporating more sophisticated, constructed covariates, e.g. kernel smoothed versions of more obvious or directly measurable variables, may facilitate such modelling. Note that such parametric models can automatically resolve the issue of consistency across different timescales, especially if formulated in continuous time, so are important even if the utilisation distribution *per se* is not of interest.

As a simple example, the Ornstein-Uhlenbeck (OU) process—see Sect. 3 below for details and references—is generally seen as a model of home range behaviour rather than selection as such, but nevertheless can be thought of as exhibiting selection in the sense of preference for locations near to a centre of attraction. It is a continuous-time model for which both the utilisation distribution and the exact conditional distribution $p(\textbf{x}(t+\delta t)|\textbf{x}(t))$ are known analytically. As a result, patterns of movement on *any* timescale can be described in terms of the parameters of the model, in a way that is automatically consistent. From a practical point of view, those parameters can be estimated from observed locations at any mixture of time intervals.

In a crucial first step towards a general approach, Michelot, Blackwell and Matthiopoulos (2019a) introduced a formal analogy between an animal moving within a utilisation distribution over ‘real’ geographical space and a Markov chain Monte Carlo algorithm which can be viewed as a process exploring a target distribution, typically the posterior distribution in some Bayesian statistical analysis, over a parameter space. This enabled the exploitation of ideas from Markov chain Monte Carlo (MCMC) statistical methods, both directly and as an inspiration for new models.

In reality this connection is not specific to MCMC; fundamentally the analogy is between an animal’s movement process and utilisation distribution and a Markov process’s transition kernel and stationary distribution. Nevertheless, the MCMC perspective has been and continues to be a very fertile way of constructing processes with known stationary distributions.

In the main part of this paper, I will look at models in discrete time briefly in Sect. 2, and then those in continuous time—both diffusion models (Sect. 3) and piecewise deterministic Markov processes (PDMPs) including velocity-jump models (Sect. 4). I will then look at extensions to movement models involving behavioural switching and interactions between individuals, in Sects. 5 and 6 respectively. Sects. 3 and 4 include both new and existing models; the modelling in Sects. 5 and 6 is new. §7 contains some brief comments on statistical issues in applying these models, with more general discussion in Sect. 8.

All computation in the paper is carried out using R (R Core Team 2024); details are given in Appendix C.

## Discrete-time models

A vast range of discrete-time models for movement have been proposed and applied. For reviews, see Patterson et al. (2017); Hooten et al. (2017). The vast majority of step selection approaches, as outlined in Sect. 1, also treat selection as if it occurs in discrete time, even if the selection-free movement kernel is defined in continuous time. Furthermore, the majority of standard MCMC algorithms are formulated in discrete time, in particular the widespread Gibbs sampling and Metropolis-Hastings algorithms. (More recent continuous-time MCMC is discussed later in Sect. 4.) It is therefore unsurprising that the link between short-term selection, utilisation distributions and MCMC was first formulated in a discrete-time context. In this section, I will revisit existing work of this kind, as the most natural way to introduce the detailed arguments.

A simple discrete-time movement model would be a random walk,

$$ \textbf{x}_{j+1} = \textbf{x}_j + \textbf{z}_{j+1}, $$

where $\textbf{x}_j$ is the position after the *j*th step and the $\textbf{z}_j$ are independent random variables from some distribution centred on zero and typically isotropic. In the terminology of §1, the movement kernel can be written as

$$ {\phi (\textbf{x}_{j+1}|\textbf{x}_j)} = \phi (\textbf{x}_{j+1}-\textbf{x}_j). $$

Barnett and Moorcroft (2008) considered combining this with a selection function $w(\cdot )$, giving the very simple discrete-time separable model

$$ p(\textbf{x}_{j+1}|\textbf{x}_j) \propto \phi (\textbf{x}_{j+1}-\textbf{x}_{j})\cdot w(\textbf{x}_{j+1}), $$

and showed that that led to a utilisation distribution

$$ \pi (\textbf{x}) \propto w(\textbf{x})\xi (\textbf{x}), $$

where

$$ \xi (\textbf{x}) = \int \! w(\textbf{z})\phi (\textbf{z}|\textbf{x}){{\text {d}}\textbf{z}}, $$

and crucially not to

$$ \pi (\textbf{x}) \propto w(\textbf{x}). $$

That is, moving locally and selecting positions according to some nominal selection function does not result in locations distributed according to that function.

In MCMC, a Metropolis-Hastings random walk algorithm is given as follows:

- given a value $\textbf{x}_j$, generate a nearby point $\textbf{x}'$ from symmetric $\psi (\textbf{x}'|\textbf{x}_j)$;
- with probability $\min \{1,\pi (\textbf{x}')/\pi (\textbf{x})\}$, accept $\textbf{x}'$ as the next value and set $\textbf{x}_{j+1}=\textbf{x}'$; otherwise reject $\textbf{x}'$ and take the next value to just be $\textbf{x}_j$ again, setting $\textbf{x}_{j+1}=\textbf{x}_j$.

If $\textbf{x}_j$ has distribution defined by $\pi (\cdot )$, then so does $\textbf{x}_{j+1}$; even if not, the distribution of $\textbf{x}_j$ approaches $\pi (\cdot )$, regardless of $\textbf{x}_0$, under very general conditions (see e.g. Gelman et al. 2014).

This algorithm would not really make sense as a movement model even in discrete time, because of the rejections. (It is perfectly possible to have moves of zero length, even with positive probability if $\psi (\cdot |\textbf{x})$ has an atom at $\textbf{x}$, but not biologically sensible for them to follow the rejection probability of the Metropolis-Hastings algorithm.) Instead, Michelot et al. (2019a) construct $\textbf{x}_{j+1}$ via a rejection-free two-stage process, in terms of some kernel $\psi (\cdot |\cdot )$. The kernel is assumed to be symmetric, $\psi (\textbf{y}|\textbf{x}) = \psi (\textbf{x}|\textbf{y})$, and spatially homogeneous, $\psi (\textbf{y}|\textbf{x}) = \psi (\textbf{y}-\textbf{x})$, and in practice will also be isotropic. Then the movement process is:

- select $$\textbf{z}$$ from $$\psi (\textbf{z}|\textbf{x}_j)$$;
- then sample $\textbf{x}_{j+1}$ from $\psi (\textbf{x}_{j+1}|\textbf{z})\pi (\textbf{x}_{j+1})$.

It can be shown that, under very weak conditions, this process has $\pi (\cdot )$ as its stationary distribution. This is known as the ‘local Gibbs’ model; for details of the connection to conventional Gibbs sampling, see the appendix to Michelot et al. (2019a). The local Gibbs model gives a simple but flexible discrete-time movement model with a reasonably simple description of individual steps, for example by choosing some parametric family for $\psi (\cdot )$, while having the specified utilisation distribution $\pi (\cdot )$. The intermediate point $\textbf{z}$ does not have any direct biological interpretation, being an artefact of the mathematical construction, but the resulting rejection-free conditional distribution for $\textbf{x}_{j+1}|\textbf{x}_j$ is more realistic than a Metropolis-Hastings random walk.

In comparing the local Gibbs model and the result of Barnett and Moorcroft (2008), note that in the absence of selection, the latter’s movement kernel $\phi$ is given by $\psi \circ \psi$, the convolution of two of the local Gibbs movement kernels. So with some abuse of notation we can summarize the results as:

*Barnett and Moorcroft:* always move according to $w\cdot \phi$, giving $\pi \propto w\cdot \xi$;

*Michelot, Blackwell, and Matthiopoulos:* alternately move according to $\psi$ and $\psi \cdot w$, giving $\pi \propto w$.

Note that in the latter case, it is only the locations after the $\psi \cdot w$ move that come from the distribution *w* and have any physical interpretation

The local Gibbs model has some limitations. Its formulation in discrete rather than continuous time has the usual drawbacks, although those are partially addressed in a pragmatic way in §2.4.3 of Michelot et al. (2020). It is computationally expensive. The modelled movement process is simplistic, having no persistence or autocorrelation in velocity; while it is possible to incorporate such persistence in discrete-time models, I prefer to address that by way of a continuous-time formulation, in Sects. 3 and 4.

## Diffusion models

In continuous time, the most obvious type of movement model would be a diffusion process on $\mathbb {R}^{d}$, the simplest example being Brownian motion. The Ornstein-Uhlenbeck (OU) process, which can be thought of as Brownian motion with an added attraction term towards a single central location, has long been used as a movement model with a well-defined utilisation distribution, albeit with a simple bivariate Gaussian form (Dunn and Gipson 1977). It can be extended to a much more flexible model, allowing for a multi-modal utilisation distribution, in two different ways, either by incorporating behavioural switching (further discussed in Sect. 5) between OU processes (Blackwell 1997, 2003) or by constructing a stochastic differential equation (SDE) with a mixture of bivariate Gaussians as its stationary distribution (Preisler et al. 2013; Gloaguen et al. 2018).

### The Langevin diffusion

To define a diffusion process with a more general stationary distribution, the simplest approach uses a Langevin diffusion process. In stochastic differential equation form, it can be written

5 $$\begin{aligned} {{\text {d}}\textbf{x}}(t) = b(\textbf{x}(t)){{\text {d}}t}+ \sqrt{\gamma }{{\text {d}}\textbf{W}(t)} \end{aligned}$$

where ${\textbf{W}_{t}}$ represents a *d*-dimensional Wiener process, and taking

$$\begin{aligned} b(\textbf{x}) = \frac{\gamma }{2}\nabla \log (\pi (\textbf{x})) \end{aligned}$$

ensures that the long-run distribution of $\textbf{x}(t)$ is $\pi (\textbf{x})$. It is convenient to write $U(\textbf{x}) = -\log (\pi (\textbf{x}))$, and so the drift term may be written as

$$\begin{aligned} b(\textbf{x}) =- \frac{\gamma }{2}\nabla U(\textbf{x}). \end{aligned}$$

In particular, to get a stationary distribution of the form of Eq. (1) requires

$$\begin{aligned} b(\textbf{x}) = \frac{\gamma }{2}\sum _{k=1}^K\beta _k\nabla c_k(\textbf{x}). \end{aligned}$$

The parameter $\gamma$ controls the speed of movement, but does not affect the stationary distribution. Thus the Langevin SDE defines a 1-parameter family of movement models with a common utilisation distribution. This approach was applied in Michelot et al. (2019b), with both the selection parameters and the speed parameter $\gamma$ estimated from location data for Steller sea lions. It was also used to combine movement and spatial survey data by Blackwell and Matthiopoulos (2024), with a refinement to correct bias; see Sect. 7.

The Langevin diffusion also defines a Markov Chain Monte Carlo algorithm. A time-discretized version of the SDE above is used to define the proposal distribution in a Metropolis-Hastings algorithm, with an accept/reject step simply to correct for the effects of the discretization. The result is the Metropolis-adjusted Langevin algorithm (MALA) (Roberts and Tweedie 1996), and the connection with the application to movement modelling is further discussed by Michelot et al. (2019b).

The Langevin movement model also relates to the analysis in Moorcroft and Barnett (2008). They start with a local selection function $w(\cdot )$ and a movement kernel $\phi _\tau (\cdot )$ that depends on the time step $\tau$. They show that taking the limit as $\tau \rightarrow 0$ of local steps following $w\cdot \phi _\tau$ gives an advection-diffusion equation, with advection/drift term involving $\nabla \log (w)$, which can be solved to give $\pi (\textbf{x}) \propto w^2(\textbf{x})$. That is:

*Moorcroft and Barnett:* moving with local drift $\nabla \log (w)$ gives $\pi \propto w^2$;

*Langevin:* moving with local drift $\nabla \log (w)/2$ gives $\pi \propto w$.

### More general diffusion models

While flexible in terms of the possible utilisation distributions, Langevin-based models have some limitations in the movement behaviour that they can represent. In particular, they have no persistence in the velocity of movement, except as induced by the shape of the utilisation distribution; where the utilisation distribution is fairly flat, the movement will be close to Brownian motion. This limits the realism of the movement patterns that can be represented.

Conversely, a number of more realistic velocity-based diffusion models exist that have stationary distributions for velocity but not for location, for example the models of Johnson et al. (2008a), Parton and Blackwell (2017) and Michelot and Blackwell (2019).

To combine both persistence in velocity and the targetting of a specified utilisation distribution, one option is the kinetic (or second order, or underdamped) Langevin diffusion (Cheng et al. 2018) which has been widely used as the basis of an MCMC algorithm, and more recently as a movement model (Michelot and Hanks 2025). It again uses only the derivative of the logarithm of the utilisation distribution, $\nabla \log (\pi (\textbf{x}))$, and is defined by linked SDEs for position $\textbf{x}$ and velocity $\textbf{v}$:

6 $$\begin{aligned} {{\text {d}}\textbf{x}}(t)&= \textbf{v}(t){{\text {d}}t}\nonumber \\ {{\text {d}}\textbf{v}}(t)&= - \frac{\gamma }{2}\nabla U(\textbf{x}(t)){{\text {d}}t}-\frac{\alpha }{\gamma }\textbf{v}(t){{\text {d}}t}+ \sqrt{\alpha }{{\text {d}}\textbf{W}(t)}. \end{aligned}$$

It gives a two-parameter family of movement models adapted to any given utilisation distribution, with the parameters allowing control over both the speed and the smoothness of the movement. In the absence of selection, it reduces to the so-called continuous-time correlated random walk (that is, integrated OU process) of Johnson et al. (2008a). An alternative model incorporating both persistence and selection is derived in §3.2.3 below.

More flexibly, Ma et al. (2019) gives *generic* machinery to build a whole range of diffusion processes adapted to a given target. Ma et al. ’s key result (their Eq. (3) and Theorem 1) is that *any* diffusion model for $\textbf{z}\in \mathbb {R}^{d_\textbf{z}}$ with target distribution $\pi _\textbf{z}(\textbf{z})$ can be written in the form

7 $$\begin{aligned} {{\text {d}}\textbf{z}}&= \left[ -\left( \textbf{D}(\textbf{z}) + \textbf{Q}(\textbf{z})\right) \nabla H(\textbf{z})+\Gamma (\textbf{z})\right] {{\text {d}}t}+ \sqrt{2\textbf{D}(\textbf{z})}{{\text {d}}\textbf{W}(t)},\nonumber \\ \Gamma _i(\textbf{z})&= \sum _{j=1}^{d_\textbf{z}} \frac{\partial }{\partial \textbf{z}_j}\left( \textbf{D}_{ij}(\textbf{z})+\textbf{Q}_{ij}(\textbf{z})\right) , \end{aligned}$$

where $H(\textbf{z}) = -\log (\pi _\textbf{z}(\textbf{z}))$, $\textbf{D}(\textbf{z})$ is a positive semi-definite matrix representing the reversible part of the process, and $\textbf{Q}(\textbf{z})$ is skew-symmetric and represents the irreversible part.

In the context of movement modelling, the variable $\textbf{z}$ can represent position, velocity, and even additional state variables, giving enormous flexibility. For example, a model that is acceleration-based rather than velocity-based could be defined by setting $\textbf{z}= (\textbf{x}, \textbf{v}, {\textbf {a}})^{\text {T}}$, where ${\textbf {a}}$ represents an acceleration vector. Similarly, Johnson et al. (2008a) use two different velocity terms in one of their examples, albeit without any stationary distribution for location; that model could be adapted to have a stationary distribution by applying the structure of Ma et al. (2019) with $\textbf{z}= (\textbf{x}, \textbf{v}_1, \textbf{v}_2)^{\text {T}}$. However, those cases are rather specialized. To generate realistic and parsimonious general-purpose movement models, it is useful to impose some restrictions. Here I will concentrate on position-based and velocity-based models, mainly with isotropic diffusion.

#### Position-based diffusion models

In a position-based model, we take $\textbf{z}\equiv \textbf{x}$, and then the simplest choices for $\textbf{D}(\cdot )$ and $\textbf{Q}(\cdot )$ are to take $\textbf{Q}\equiv \textbf{0}$ and take $\textbf{D}$ to be constant and isotropic,

$$ \textbf{D}(\textbf{x}) = {\textstyle \frac{{1}}{{2}}}\gamma I_d, $$

where $I_d$ denotes the $d\times d$ identity matrix. This leads to the first order Langevin model of Eq. (5). Alternatively, we can allow the diffusion rate to depend on position; it seems most natural to do that in an isotropic way, with

$$ \textbf{D}(\textbf{x}) = {\textstyle \frac{{1}}{{2}}}\tau (\textbf{x}) I_d $$

say, and to retain $\textbf{Q}\equiv \textbf{0}$. Then from Eq. (7), $\Gamma (\textbf{x}) = {\textstyle \frac{{1}}{{2}}}\nabla \tau (\textbf{x})$, and

$$\begin{aligned} {{\text {d}}\textbf{x}}(t) = {\textstyle \frac{{1}}{{2}}}[\nabla \tau (\textbf{x})-\tau (\textbf{x})\nabla U(\textbf{x})]{{\text {d}}t}+ \sqrt{\tau (\textbf{x})}{{\text {d}}\textbf{W}(t)}\end{aligned}$$

also has utilisation distribution $\pi (\textbf{x})$. In general, the diffusion rate could depend on covariates directly, with

8 $$\begin{aligned} \tau (\textbf{x})&= \exp \left( \delta _0+ \sum _{k=1}^K\delta _k c_k(\textbf{x})\right) \end{aligned}$$

say. The covariates can be arranged to be the same ones that influence the utilisation distribution in Eq. (1), if necessary taking some coefficients $\beta _k$ or $\delta _k$ to be zero. As a special case, we could take the diffusion rate to be inversely proportional to the utilisation distribution,

9 $$\begin{aligned} \tau (\textbf{x})&\propto 1/\pi (\textbf{x}), \end{aligned}$$

by setting $\delta _k = -\beta _k$ for $k=1,\ldots ,K$. Since it is often more convenient if we need to know $\pi (\textbf{x})$ only up to proportionality, we can write this as

10 $$\begin{aligned} \tau (\textbf{x})&= {\alpha }^2/ \exp \left( \sum _{k=1}^K \beta _k c_k(\textbf{x})\right) \end{aligned}$$

11 $$\begin{aligned}&= {\alpha }^2/\exp ({L}(\textbf{x})) \end{aligned}$$

which simplifies to give

12 $$\begin{aligned} {{\text {d}}\textbf{x}}(t) = {\alpha }\exp (-{\textstyle \frac{{1}}{{2}}}{L}(\textbf{x})){{\text {d}}\textbf{W}(t)}. \end{aligned}$$

That is, the diffusion rate depends on $\pi (\textbf{x})$, and there is *no* drift term i.e. no directed movement. In biological terms, this represents a case where an animal attains its utilisation distribution simply by moving more quickly in less desirable regions, without regard to its direction of movement.

**Fig. 1:**
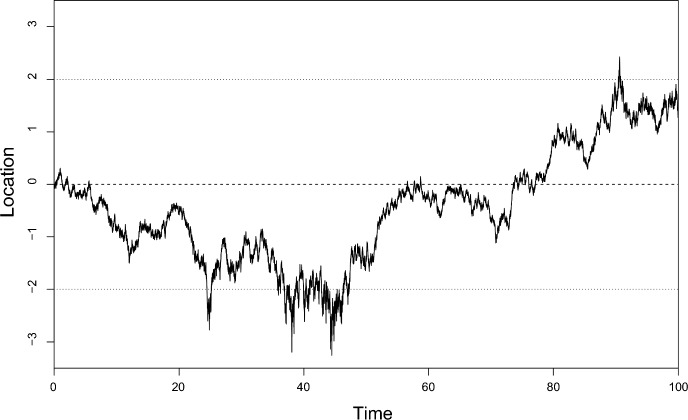
A movement process in 1 dimension with diffusion rate inversely proportional to the density of the target distribution, i.e. of the utilisation distribution of the animal, following Eq. (12). In this example, the utilisation distribution is a standard normal distribution. The horizontal dashed line at zero indicates the centre and peak of the utilisation distribution; the horizontal dotted lines at $\pm 2$ enclose approximately 95% of the integrated utilisation distribution. For computational details, see Appendix C.1.

An example of a very simple case is shown in Fig. 1. The environment here is 1-dimensional, since the variation in diffusion rate is not readily apparent when plotting a trajectory in 2 dimensions, though of course it would be detectable statistically. The underlying utilisation distribution i.e. the target distribution is simply the standard normal distribution, again for simplicity of illustration. The dependence of the diffusion rate on location is clear, especially in the excursions below $-2$ at around time 40.

#### Velocity-based diffusion models

In a velocity-based model with $\textbf{z}= (\textbf{x}, \textbf{v})^{\text {T}}$, it is natural to write ${{\text {d}}\textbf{z}}= ({{\text {d}}\textbf{x}}, {{\text {d}}\textbf{v}})^{\text {T}}$ and to require

13 $$\begin{aligned} {{\text {d}}\textbf{x}}= \textbf{v}{{\text {d}}t}. \end{aligned}$$

Many different forms of model are possible under that constraint. A flexible but plausible class is given as follows. The velocity has a circular normal distribution, with a variance that depends on location,

$$ \textbf{v}|\textbf{x}\sim {\text {Normal}}(\textbf{0}, h(\textbf{x})I_d), $$

and the diffusion of velocity is isotropic,

$$\begin{aligned} \textbf{D}(\textbf{z})&= \begin{pmatrix}\textbf{0}& \quad \textbf{0}\\ \textbf{0}& \quad a(\textbf{x})I_d\end{pmatrix}. \end{aligned}$$

Then

$$\begin{aligned} H(\textbf{z})&= U(\textbf{x}) + \frac{d}{2}\log h(\textbf{x}) + \frac{\textbf{v}^{\text {T}}\textbf{v}}{2h(\textbf{x})},\\ \nabla H(\textbf{z})&= (\nabla _\textbf{x}H(\textbf{z}), \nabla _\textbf{v}H(\textbf{z}))^{\text {T}}, \end{aligned}$$

where

$$\begin{aligned} \nabla _\textbf{x}H(\textbf{z})&= \nabla U(\textbf{x}) + \frac{d}{2}\frac{\nabla h(\textbf{x})}{ h(\textbf{x})} - \frac{\nabla h(\textbf{x})\textbf{v}^{\text {T}}\textbf{v}}{2h(\textbf{x})^2},\\ \nabla _\textbf{v}H(\textbf{z})&= \textbf{v}/h(\textbf{x}). \end{aligned}$$

To satisfy Eq. (13), the simplest choice of $\textbf{Q}$ is

$$\begin{aligned} \textbf{Q}(\textbf{z})&= \begin{pmatrix}\textbf{0}& -h(\textbf{x}) I_d\\ h(\textbf{x}) I_d& \textbf{0}\end{pmatrix}. \end{aligned}$$

Substituting into Eq. (7) then gives

$$\begin{aligned} {{\text {d}}\textbf{v}}=&\left[ -h(\textbf{x})\nabla U(\textbf{x}) + \left( 1-\frac{d}{2}\right) \nabla h(\textbf{x}) + \frac{1}{2}\nabla h(\textbf{x})\frac{\textbf{v}^{\text {T}}\textbf{v}}{h(\textbf{x})} - \frac{a(\textbf{x})}{h(\textbf{x})}\textbf{v}+ \nabla a(\textbf{x}) \right] {{\text {d}}t}\\&+ \sqrt{2a(\textbf{x})}{{\text {d}}\textbf{W}(t)}\end{aligned}$$

As a special case, the second-order Langevin model is obtained by setting

$$ a(\textbf{x}) = \alpha /2, ~ h(\textbf{x}) = \gamma /2, $$

to give

$$\begin{aligned} \textbf{D}(\textbf{z})&= \begin{pmatrix}\textbf{0}& \quad \textbf{0}\\ \textbf{0}& \quad \alpha I_d/2\end{pmatrix},\\ \textbf{Q}(\textbf{z})&= \begin{pmatrix}\textbf{0}& \quad -\gamma I_d/2\\ \gamma I_d/2& \quad \textbf{0}\end{pmatrix}, \end{aligned}$$

recovering the model of Eq. (6).

#### Polar velocity-based diffusion models

While the approach above (§3.2.2) of defining velocity in Cartesian co-ordinates seems the simplest way of obtaining a targetted velocity-based model, it is perhaps more natural to consider expressing velocity in terms of speed and bearing. This gives a straightforward way to express a continuous-time step-and-turn model in the sense of Parton et al. (2017) and Parton and Blackwell (2017), in which the animal’s bearing is changing continuously, and to adjust it to target a general utilisation distribution.

For simplicity, I will explicitly limit the formulation to two dimensions, though of course three-dimensional polar co-ordinates could be used instead. We take $\textbf{z}= (x, y, s, \theta )^{\text {T}}$, and require

14 $$\begin{aligned} {\text {d}}x&= s\cos \theta {{\text {d}}t},\nonumber \\ {\text {d}}y&= s\sin \theta {{\text {d}}t}. \end{aligned}$$

The Ma et al. (2019) framework allows great flexibility here, but one simple case is when speed has distribution $\pi _s(\cdot )$ and bearing has a uniform distribution, both independently of position and of each other. Then

$$\begin{aligned} H(\textbf{z})&= U(\textbf{x}) + H_s(s),\\ \nabla H(\textbf{z})&= \left( \frac{\partial U(\textbf{x})}{\partial x}, \frac{\partial U(\textbf{x})}{\partial y}, \frac{\partial H_s(s)}{\partial s}, 0\right) ^{\text {T}}. \end{aligned}$$

If

$$ \textbf{D}(\textbf{z}) = \begin{pmatrix} 0& \quad 0& \quad 0& \quad 0\\ 0& \quad 0& \quad 0& \quad 0\\ 0& \quad 0& \quad \gamma /2& \quad 0\\ 0& \quad 0& \quad 0& \quad \alpha /2 \end{pmatrix} $$

then the simplest form for $\textbf{Q}(\textbf{z})$ that satisfies Eqs. (14) is

$$ \textbf{Q}(\textbf{z}) = \begin{pmatrix} 0& \quad 0& \quad ~0~& \quad s\sin \theta \\ 0& \quad 0& \quad 0& \quad -s\cos \theta \\ 0& \quad 0& \quad 0& \quad 0\\ -s\sin \theta & \quad s\cos \theta & \quad 0& \quad 0 \end{pmatrix} $$

giving

15 $$\begin{aligned} {{\text {d}}s}&= -\frac{\gamma }{2}\frac{\partial H_s(s)}{\partial s}{{\text {d}}t}+ \sqrt{\gamma }{\text {d}}\textbf{W}_s(t) \end{aligned}$$

16 $$\begin{aligned} {{\text {d}}\theta }&= (s\sin \theta , -s\cos \theta )\nabla U(\textbf{x}){{\text {d}}t}+ \sqrt{\alpha }{\text {d}}\textbf{W}_\theta (t). \end{aligned}$$

Note that Eq.(15) does not depend on the target distribution, and is just a 1-dimensional Langevin SDE for *s*, and that the drift coefficient in Eq. (16) is the negative perpendicular dot product of velocity and $\nabla U$. An appealing choice for $\pi _s(\cdot )$, giving a circular bivariate Normal distribution for velocity and so allowing straightforward comparison with other models, is

$$\begin{aligned} s&\sim {\text {Rayleigh}}(\sigma ),\\ \pi _s(s)&= \frac{s}{\sigma ^2}\exp \left( -\frac{s^2}{2\sigma ^2}\right) ,\\ H_s(s)&= \frac{s}{\sigma ^2}-\frac{1}{s}. \end{aligned}$$

Alternatively, speed may be taken to be constant, particularly in combination with switching between behaviours (see §5) which each have a different speed.

More generally, the polar velocity approach, in combination with the general representation of Ma et al. (2019), would allow a targetted movement process with a preference for moving in particular directions, dependent on location; Munden et al. (2021), discussed in §1, give an example where goats exhibit such a preference. Modelling choices at this level of detail are unavoidably application-specific, so are not pursued here.

**Fig. 2:**
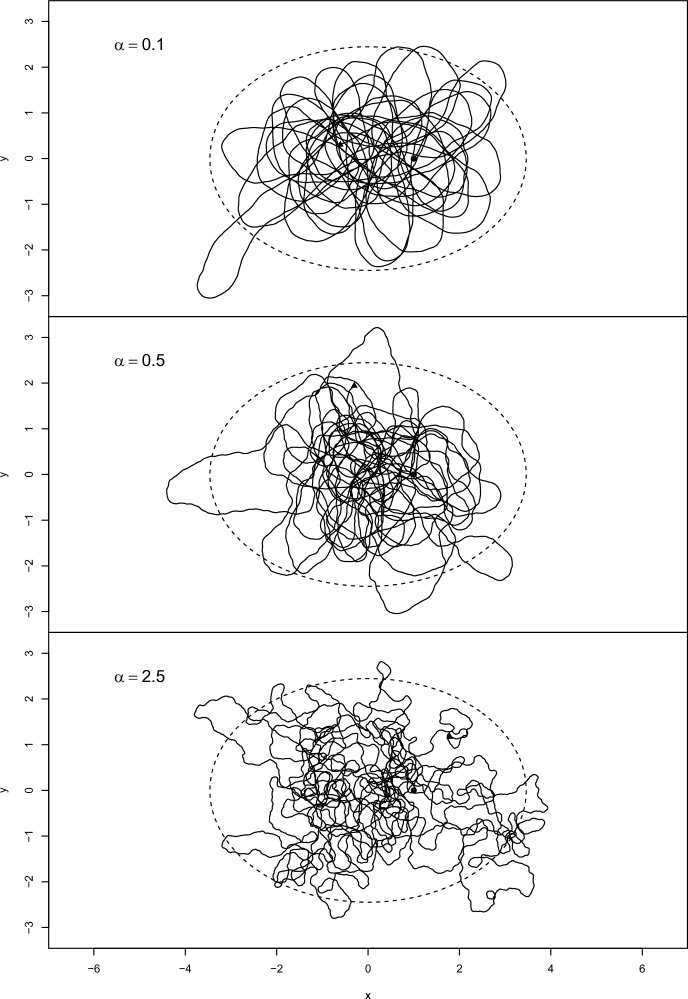
Polar velocity models in a 2-dimensional environment, as defined in Eqs. (15) and (16), with constant speed ($\gamma =0$) and with the diffusion coefficient $\alpha$ on the bearing varying as shown; all other properties are identical in each simulation. In each case, the solid line represents a movement trajectory, with solid circles and triangles indicating starts and ends respectively, and the dashed line represents a contour of the bivariate normal target distribution, $$U(\textbf{x}) \propto \exp (-{\textstyle \frac{{1}}{{2}}}\textbf{x}^{\text {T}}\big ({\begin{smallmatrix} 2 & 0\\ 0 & 1 \end{smallmatrix}}\big )^{-1} \textbf{x})$$. For computational details, see Appendix C.2

To illustrate these models, Figure 2 shows realisations of models defined by Eq. (16) in which the speed *s* is constant; that is, $\gamma =0$ in Eq. (15), which therefore simply reduces to ${{\text {d}}s}=0$. In each case, the target distribution for $\textbf{x}$ is just a bivariate normal distribution, with $${\pi }(\textbf{x}) \propto \exp (-{\textstyle \frac{{1}}{{2}}}\textbf{x}^{\text {T}}\big ({\begin{smallmatrix} 2 & 0\\ 0 & 1 \end{smallmatrix}}\big )^{-1} \textbf{x})$$. The cases shown differ only in the value of $\alpha$, the diffusion coefficient of the bearing; from top to bottom, these are $\alpha = 0.1, 0.5, 2.5$.

Visually, a key feature of these paths is the difference in sinuosity or ‘wiggliness’ between them. Sinuosity is an important property of paths in movement ecology both theoretically (Benhamou 2004) and practically (e.g. Nguyen et al. 2024), and in the absence of selection would correspond closely to the parameter $\alpha$. Exploring the connection between $\alpha , \pi (\textbf{x})$ and empirical measures of sinuosity would be of interest, but is beyond the scope of the current paper.

## Piecewise deterministic Markov processes

A piecewise deterministic Markov process (PDMP) is a continuous-time process that evolves in a deterministic way except at a discrete set of (possibly random) times $\ldots , t_1, t_2, t_3,\ldots$, at which its state changes in some random way. Davis (1993) gives a wide range of examples, and many continuous-time MCMC algorithms fall into this category, including Hamiltonian Monte Carlo (see also e.g. Gelman et al. (2014)) and various samplers described below.

For movement modelling, the primary interest is in ‘velocity-jump’ models in $\mathbb {R}^{d}$ (where usually $d=2$); these are PDMP processes for $(\textbf{x},\textbf{v})$, and so take values in $\mathbb {R}^{2d}$, where $\textbf{v}$ is constant except at a discrete set of ‘jump’ times and $\textbf{x}$ follows straight line segments determined by $\textbf{v}$. These include ‘run and tumble’ models (Othmer et al. 1988) and other models where paths are made up of line segments e.g. Codling and Hill (2005a, 2005b). Recent work includes Ruiz-Suarez et al. (2020) and Munden et al. (2021).

To use such models to target a particular distribution, we can extend the MCMC analogy to include them, starting with some relatively recent developments in MCMC literature.

### Existing samplers

The bouncy particle sampler (BPS; Peters and de With 2012; Bouchard-Côté et al. 2018; Fearnhead et al. 2018) has two types of event that change velocity: bounces and refreshment. In the context of animal movement, these correspond to changes in velocity that occur in response to selection, and those that occur completely at random, respectively.

Refreshment occurs at a constant rate, $\lambda _0$ say, and involves resampling from the velocity distribution. Bounces occur at a rate that depends on both velocity and target, and reflect the velocity in a deterministic way. The rate is given by

17 $$\begin{aligned} \lambda (\textbf{x},\textbf{v}) = \max \{0, \nabla U(\textbf{x}){\cdot }\textbf{v}\}. \end{aligned}$$

Reflection itself can be defined more formally—in a way that proves useful for more general models—as follows. When a bounce occurs at $\textbf{x}$, with current velocity $\textbf{v}$, we decompose $\textbf{v}$ into a component $\textbf{v}_1$ in the direction of the gradient $\nabla U(\textbf{x})$ and a component $\textbf{v}_2$ orthogonal to $\nabla U(\textbf{x})$ and hence to $\textbf{v}_1$, so $\textbf{v}= \textbf{v}_1 + \textbf{v}_2$. The new velocity after the bounce is given simply by

$$ \textbf{v}' = -\textbf{v}_1 + \textbf{v}_2. $$

The generalized BPS (GBPS; Changye and Robert 2017, 2020) adds some stochasticity to the bounce, as follows. The velocity is decomposed as $\textbf{v}= \textbf{v}_1 + \textbf{v}_2$, and component $\textbf{v}_1$ reflected, as before; $\textbf{v}_2$ is replaced with a randomized orthogonal term. That is,

$$ \textbf{v}' = -\textbf{v}_1 + \textbf{u}$$

where $\textbf{u}\sim {\text {Normal}}(\textbf{0},\sigma ^2I_{d-1})$ is defined on the subspace orthogonal to $\nabla U(\textbf{x})$ and $\textbf{v}_1$, denoted by $\Omega _2 = \left\{ \nabla U(\textbf{x})\right\} ^\perp$.

For the GBPS, refreshment events are no longer necessary to ensure irreducibility; for movement modelling, they remain useful and will be retained throughout.

### The half-space bouncy particle sampler

Additional flexibility in the way velocities change is needed for movement modelling, since it is unrealistic to insist that a particular component of the animal’s velocity is reflected exactly. To address this, the broader formulation of Fearnhead et al. (2018) is useful. Specifically, the rate of bounce events and the distribution of updated velocities, $q(\textbf{v}'|\textbf{x},\textbf{v})$ need to satisfy their Eq. (6) to ensure that the stationary distribution is preserved. In the present section, the rate of bounce events given in Eq. (17), which Fearnhead et al. (2018) call the canonical rate, is retained, and $q(\textbf{v}'|\textbf{x},\textbf{v})$ is varied. In §4.3, the assumption of canonical rates will be relaxed too. In each case, the motivation is to give enough flexibility for realistic movement modelling and more specifically to extend the support of the distribution $q(\textbf{v}'|\textbf{x},\textbf{v})$.

From Eq. (6) of Fearnhead et al. (2018), if the canonical bounce rate is retained then there are two obvious cases to consider: $\nabla U(\textbf{x}){\cdot }\textbf{v}> 0$ and $\nabla U(\textbf{x}){\cdot }\textbf{v}\le 0$.

In the former case, the first term in their (6) equals the RHS, so the integral term must be zero. So for almost all $\textbf{v}'$ such that $p(\textbf{v}')>0$, either $\lambda (\textbf{x},\textbf{v}')=0$ or $q(\textbf{v}|\textbf{x},\textbf{v}')=0$. Given the form of $\lambda$, that means that

$$ \nabla U(\textbf{x}){\cdot }\textbf{v}> 0, \nabla U(\textbf{x}){\cdot }\textbf{v}' > 0 \implies q(\textbf{v}|\textbf{x},\textbf{v}')=0. $$

That is, with canonical bounce rates, the component of velocity parallel to $\nabla U(\textbf{x})$ must change sign at a bounce. So for any $\textbf{v}$, the support for $\textbf{v}'$ is at most the half-space—if $d=2$, the half-plane—defined by that condition. In practice, the model derived will have support for $\textbf{v}'$ equal to all of that half-space.

Allowable forms for $q(\textbf{v}'|\textbf{x},\textbf{v})$ (over its support) are obtained by considering Fearnhead et al. ’s (6) in the case $\nabla U(\textbf{x}){\cdot }\textbf{v}\le 0$. Then the first term is zero, and we know that only values of $\textbf{v}'$ with $\nabla U(\textbf{x}){\cdot }\textbf{v}' > 0$ have non-zero $\lambda (\textbf{x},\textbf{v}')$ so we require

$$ -\int _{\textbf{v}' \in \Omega ^+} \nabla U(\textbf{x}){\cdot }\textbf{v}' q(\textbf{v}|\textbf{x},\textbf{v}') p(\textbf{v}') {\text {d}}\textbf{v}' = p(\textbf{v}) \nabla U(\textbf{x}){\cdot }\textbf{v}$$

where $\Omega ^+$ is the half-space of $\textbf{v}'$ such that $\nabla U(\textbf{x}){\cdot }\textbf{v}' > 0$.

We can decompose a velocity $\textbf{v}$ at $\textbf{x}$ as $\textbf{v}= \textbf{v}_1+\textbf{v}_2$ where $\textbf{v}_1, \textbf{v}_2$ are parallel to and perpendicular to $\nabla U(\textbf{x})$ respectively, taking values in $\Omega _1 = \left\{ \nabla U(\textbf{x})\right\}$ and $\Omega _2 = \left\{ \nabla U(\textbf{x})\right\} ^\perp$ as before. If $\textbf{v}$ has an isotropic multivariate normal distribution ${\text {Normal}}(\textbf{0},\sigma ^{2} I_2)$, we can write

$$ p(\textbf{v}) = f_1(\textbf{v}_1)f_2(\textbf{v}_2) $$

where $f_1(\cdot )$ is the univariate ${\text {Normal}}(0,\sigma ^2)$ density and $f_2(\cdot )$ is the multivariate ${\text {Normal}}(\textbf{0},\sigma ^2I_{d-1})$ density on $\Omega _2$. Motivated by the BPS and GBPS, we can also decompose *q* similarly and write

$$ q(\textbf{v}'|\textbf{v}) = q_1(\textbf{v}_1'|\textbf{x},\textbf{v}_1)q_2(\textbf{v}_2'|\textbf{x},\textbf{v}_2). $$

From Fearnhead et al. ’s Eq. (6) in this case we then require

$$\begin{aligned}&-\int _{\textbf{v}_1'\in \Omega _1^+}\int _{\textbf{v}_2'}\nabla U(\textbf{x})\cdot \textbf{v}_1' q_1(\textbf{v}_1|\textbf{x},\textbf{v}_1')q_2(\textbf{v}_2|\textbf{x},\textbf{v}_2') f_1(\textbf{v}_1')f_2(\textbf{v}_2') {\text {d}}\textbf{v}_1'{\text {d}}\textbf{v}_2'\\ =&~ f_1(\textbf{v}_1)f_2(\textbf{v}_2) \nabla U(\textbf{x})\cdot \textbf{v}_1. \end{aligned}$$

A natural sufficient condition is that

$$\begin{aligned} \int _{\textbf{v}_1'\in \Omega _1^+} \nabla U(\textbf{x})\cdot \textbf{v}_1' q_1(\textbf{v}_1|\textbf{x},\textbf{v}_1') f_1(\textbf{v}_1'){\text {d}}\textbf{v}_1'&=\nabla U(\textbf{x})\cdot \textbf{v}_1' f_1(\textbf{v}_1),\\ \int _{\textbf{v}_2'} q_2(\textbf{v}_2|\textbf{x},\textbf{v}_2') f_2(\textbf{v}_2'){\text {d}}\textbf{v}_2'&= f_2(\textbf{v}_2). \end{aligned}$$

We can then define $q_1$ and $q_2$ separately.

For $q_1$, note that by definition $\textbf{v}_1,\textbf{v}_1'$ are parallel to $\nabla U(\textbf{x})$, and can be written as $s_1\textbf{u}, s_1'\textbf{u}$ respectively, where $\textbf{u}=\nabla U(\textbf{x})/||\nabla U(\textbf{x})||$. Then the condition on $q_1(\cdot )$ becomes

$$\begin{aligned} \int _{s_1'\ge 0} s_1'q_1(s_1|\textbf{x},s_1')f_1(s_1'){\text {d}}s_1' = s_1f_1(s_1). \end{aligned}$$

The occurrence of $s_1 f_1(s)$, where $f_1(\cdot )$ represents ${\text {Normal}}(0,\sigma ^2)$, suggests a form based on the Rayleigh distribution. Since $s_1,s_1'$ have opposite signs, define $r_1 = -s_1'$ and let $(s_1,r_1)$ have a correlated bivariate Rayleigh distribution (Mallik 2000) with equal Rayleigh marginals with scale parameter $\sigma$, and a non-negative correlation parameter $\rho$. That is,

$$ p(s_1,r_1) = \frac{s_1 r_1}{\sigma ^{{4}}(1-\rho ^2)} \exp \left( -\frac{s_1^2+r_1^2}{2\sigma ^2(1-\rho ^2)}\right) I_0\left( {\frac{\rho s_1 r_1}{\sigma ^2(1-\rho ^2)}}\right) $$

where $I_0(\cdot )$ is the zeroth-order modified Bessel function of the first kind. Then define $q_1$ as the corresponding conditional distribution, which is the Rice distribution with scale parameter $\sqrt{\sigma ^2(1-\rho ^2)}$ and non-centrality parameter $-s_1\rho$:

$$ s_1'|\textbf{x},s_1 \sim {\text {Rice}}(-s_1\rho ,\sqrt{\sigma ^2(1-\rho ^2)}) $$

or

$$ q_1(s_1'|\textbf{x},s_1 ) = \frac{-s_1}{\sigma ^2(1-\rho ^2)}\exp \left( \frac{(s_1')^2+(s_1\rho )^2}{2\sigma ^2(1-\rho ^2)}\right) I_0\left( \frac{-s_1's_1\rho }{\sigma ^2(1-\rho ^2)}\right) ,~~s_1'\ge 0. $$

See Rice (1945) and also the R package VGAM (Yee 2024). Note that both the BPS and GBPS correspond to taking $\rho =1$, so that the speeds parallel to the direction of the gradient, before and after the bounce, are perfectly correlated. Full support on the half-space of velocities requires $0\le \rho <1$.

For $q_2$, the integral equation simply amounts to maintaining detailed balance for the velocity perpendicular to the gradient, with respect to its stationary distribution. A convenient general form is to take $(\textbf{v}_2,\textbf{v}_2')$ to have a symmetric correlated multivariate normal distribution,

18 $$\begin{aligned} (\textbf{v}_2,\textbf{v}_2') \sim {\text {Normal}}(\textbf{0},\sigma ^2 \left( \begin{matrix}I_{d-1}& \omega I_{d-1}\\ \omega I_{d-1}& I_{d-1}\end{matrix}\right) ), \end{aligned}$$

so that $q_2$ is the corresponding conditional distribution and

$$ \textbf{v}_2'|\textbf{x},\textbf{v}_2 \sim {\text {Normal}}(\omega \textbf{v}_2,(1-\omega ^2)\sigma ^2 I_{d-1}). $$

Effectively, the changes in orthogonal velocity, when bounces occur, are autoregressive. The BPS corresponds to $\omega =1$, and the GBPS to $\omega =0$; setting $\omega =-1$ gives what Fearnhead et al. (2018) call the Pure Reflection case. Full support on the half-space of velocities requires $-1<\omega <1$; for movement modelling, it seems natural to further require $0\le \omega <1$.

So at a ‘bounce’ event, the half-space sampler produces a new velocity consisting of a speed in the direction of the gradient that has a Rice distribution and a speed orthogonal to the gradient that has a Normal distribution; in each case, the speed is correlated with the speed in that direction before the bounce.

### Velocity updates with full support

Relaxing the assumption of canonical rates defined by Eq. (17), it is possible to have velocity updates that occur at a rate $\lambda (\textbf{x},\textbf{v})$ that is a smooth function of $\textbf{x}$ and $\textbf{v}$ (and strictly positive almost everywhere), and for which the velocity after the update has full support in the space of velocities. It is counter-intuitive to refer to these as ‘bounces’, since there is no restriction on the direction of movement before or after; instead I will call them ‘reorientations’. A general constructive approach, as for diffusions, is not yet available, but this section shows a range of simple tractable examples with qualitatively different properties.

For this purpose, it is convenient to express velocities in polar co-ordinates, as was done in §3.2.3 for diffusion models. Again, I will focus on the 2-dimensional case for simplicity, but extension to higher dimensions would be straightforward if necessary. For a velocity $\textbf{v}$, denote the corresponding speed by $s= ||\textbf{v}|| = \sqrt{v_x^2+v_y^2}$ and the bearing or direction of movement by $\arg (\textbf{v}) = \tan _2^{-1}(v_y,v_x)$, where $\tan _2^{-1}(\cdot ,\cdot )$ denotes the 2-argument inverse tangent function, and $v_x, v_y$ are the components of $\textbf{v}$ expressed in Cartesian co-ordinates. Assuming the distribution of $\textbf{v}$ to be isotropic, i.e. the direction has a uniform distribution on $(-\pi ,\pi ]$, we then write

$$ p(\textbf{v}) = \frac{1}{2\pi }p(s). $$

To obtain a movement model consistent with a given target utilisation distribution, it is necessary to satisfy Eq. (6) of Fearnhead et al. (2018) at any given point $\textbf{x}$. It is convenient to consider bearing relative to the direction of $\nabla U(\textbf{x})$,

$$ \theta (\textbf{x},\textbf{v}) = \arg (\textbf{v})-\arg (\nabla U(\textbf{x})), $$

or simply $\theta$. Motivated by existing results, I will also write

$$ \kappa (\textbf{x},\theta ) = \frac{\lambda (\textbf{x},\textbf{v})}{s||\nabla U(\textbf{x})||}. $$

To obtain some illustrative example models, I will assume that speed and bearing are updated independently at a reorientation, with

$$ q(\textbf{v}'|\textbf{x},\textbf{v}) = q(s'|s)q(\theta '|\textbf{x},\theta ). $$

Then Fearnhead et al. (2018)’s Eq. (6) becomes

$$\begin{aligned} \frac{1}{2\pi }p(s)s||\nabla U(\textbf{x})||\kappa (\textbf{x},\theta )&- \int _{-\pi }^{\pi }\int _0^{\infty }s'||\nabla U(\textbf{x})||\kappa (\textbf{x},\theta ') q(s|s')q(\theta |\textbf{x},\theta ')\frac{1}{2\pi }p(s'){{\text {d}}s}'{{\text {d}}\theta }'\\&= \frac{1}{2\pi }p(s)s||\nabla U(\textbf{x})||\cos (\theta ), \end{aligned}$$

cancelling to

19 $$\begin{aligned}&p(s)s\kappa (\textbf{x},\theta ) - \int _{-\pi }^{\pi }\int _0^{\infty }s'\kappa (\textbf{x},\theta ') q(s|s')q(\theta |\textbf{x},\theta ')p(s'){{\text {d}}s}'{{\text {d}}\theta }' =p(s)s\cos (\theta ),\nonumber \\&\quad \equiv p(s)s\kappa (\textbf{x},\theta ) -\int _0^{\infty }s' q(s|s')p(s'){{\text {d}}s}' \int _{-\pi }^{\pi }\kappa (\textbf{x},\theta ')q(\theta |\textbf{x},\theta '){{\text {d}}\theta }' =p(s)s\cos (\theta ). \end{aligned}$$

Eq. (19) simplifies if $q(s'|s)$ satisfies

20 $$\begin{aligned} \int _0^{\infty }s' q(s|s')p(s'){{\text {d}}s}' = sp(s), \end{aligned}$$

and the repeated occurence of *sp*(*s*) suggests that a size-biased version of the speed distribution has a rôle here. Given a density $p(\cdot )$ on the positive real line, the corresponding size-biased density $p^*(\cdot )$ is defined by

$$ p^*(z) = zp(z)/m, $$

where *m* is the expectation of $p(\cdot )$, that is $m = \int zp(z){\text {d}}z$. We can therefore define $q(s'|s)$ by constructing a symmetric joint density $q(s,s')$ with both marginals given by the size-biased speed density $p^*(\cdot )$, and letting $q(s'|s) = q(s,s')/p^*(s)$. This satisfies Eq. (19), and includes as special cases $s'=s$ and $s'$ independent of *s*.

If $\textbf{v}$ has an isotropic multivariate normal distribution ${\text {Normal}}(\textbf{0},\sigma ^2 I_2)$, as previously, then *p*(*s*) is the ${\text {Rayleigh}}(\sigma )$ density, and $p^*(s)$ is the ${\text {Maxwell-Boltzmann}}(\sigma )$ density (also a Nakagami-*m* distribution (Nakagami 1960) with shape parameter $m=3/2$, or a scaled $\chi _3$ density)

$$ p^*(s) = \sqrt{\frac{2}{\pi }}\frac{s^2}{\sigma ^3}\exp (-s^2/2\sigma ^2). $$

The corresponding joint density, based on Nakagami (1960); Lopez-Martinez et al. (2013) is

$$\begin{aligned} q(s,s') = \frac{(3s's/2)^{3/2}}{\varsigma ^2\sigma ^3\sqrt{\pi \rho /2}} \exp \left( -\frac{(s')^2+s^2}{2\varsigma ^2}\right) )I_{\frac{1}{2}}\left( \frac{s's\rho }{\varsigma ^2}\right) , \end{aligned}$$

where $\varsigma ^2=2\sigma ^2(1-\rho ^2)/3$ , resulting in a conditional distribution to be used in the reorientation update given by a scaled non-central $\chi _3$ distribution (e.g. Kim et al. (2000); also known as a generalized Rayleigh distribution)

$$ q(s'|s) = \frac{(s')^{3/2}}{\varsigma ^2\sqrt{\rho s}}\exp \left( -\frac{(s')^2+(\rho s)^2}{2\varsigma ^2}\right) I_{\frac{1}{2}}\left( \frac{s's\rho }{\varsigma ^2}\right) . $$

When $q(s'|s)$ satisfies Eq. (20) for all *s*, Eq. (19) becomes

21 $$\begin{aligned} \kappa (\textbf{x},\theta ) - \int _{-\pi }^{\pi }\kappa (\textbf{x},\theta ')q(\theta |\textbf{x},\theta '){{\text {d}}\theta }' =\cos (\theta ). \end{aligned}$$

A completely general solution to Eq. (21) is beyond the scope of this paper, but a family of solutions that exhibits a range of qualitatively different forms is given by

22 $$\begin{aligned} \kappa (\textbf{x},\theta )&= 1 + h\cos (\theta ), \end{aligned}$$

23 $$\begin{aligned} q(\theta '|\theta )&= \frac{1}{2\pi }\left\{ 1 + a\cos (\theta ') + b \cos (\theta '-\theta )\right\} . \end{aligned}$$

Ensuring positivity of $\kappa (\textbf{x},\theta )$ and $q(\theta '|\theta )$ requires that $|h|\le 1$ and $|a|+|b|\le 1$, and solving Eq. (21), after integrating and simplifying using basic trignometric identities, requires

24 $$\begin{aligned} h - a - \frac{bh}{2} = 1. \end{aligned}$$

The space of feasible solutions is a quadrilateral region in (*a*, *b*)-space (see Appendix A for further details). The vertices of this region, plus one additional point of interest, are described in Table 1.

**Table 1:** Extremal solutions of Eq. (24). The first four rows given the vertices of the feasible region in (*a*, *b*)-space; see Appendix A for details. The final row relates to the case $a=b=0$, of interest because of its simplicity; it lies on the boundary of the feasible region

Case	*a*	*b*	*h*	Interpretation
(i)	−1/3	2/3	1	Reorientation is biased towards higher target densities and towards the current bearing; rate of reorientations increases with $\cos (\theta )$
(ii)	−1	0	0	Reorientation is biased towards higher target densities and independent of current bearing; rate of reorientations is independent of $\theta$
(iii)	0	−1	2/3	Reorientation is independent of the target density and biased away from the current bearing; rate of reorientations increases with $\cos (\theta )$
(iv)	1/3	−2/3	1	Reorientation is biased away from higher target densities and away from the current bearing; rate of reorientations increases with $\cos (\theta )$
(v)	0	0	1	Reorientation is isotropic i.e. independent of target density and current bearing; rate of reorientations increases with $\cos (\theta )$

These examples show that a process of reorientations that is consistent with a given utilisation distribution can be achieved in a variety of ways. For example, case (i) is perhaps closest to existing ‘off-the-shelf’ models, since current bearing has an important effect, while in case (v) the reorientations themselves are isotropic, and the utilisation distribution affects only their rate, to some extent echoing the diffusion model in Eq. (12). The shapes of the functions in Eqs. (22) and (23) could be completely generalized, for example by extending the cosine terms to a complete Fourier expansion, leading to more complicated constraints on the coefficients.

### Autocorrelated refreshment

For movement modelling, it is also useful to generalize the ‘refreshment’ process, as defined in §4.1, which does not depend on the target distribution. In the BPS, refreshment involves sampling from the marginal distribution for velocity, independently of current velocity. Similarly, non-targeted velocity-jump or step-and-turn processes often involve turns where the bearing has some distribution centred on the current bearing, but the speed is sampled independently of the current speed (except perhaps for dependence due to some discrete underlying behavioural process—c.f. §5). But that independence is neither necessary nor particularly natural.

There are at least two natural ways to include autocorrelation in successive velocities: defining a joint distribution directly on the velocities, or working with speeds and directions separately. If velocities are ${\text {Normal}}(\textbf{0},\sigma ^2 I_d)$, then defining

25 $$\begin{aligned} (\textbf{v},\textbf{v}') \sim {\text {Normal}}\bigg (\textbf{0},\sigma ^2 \bigg (\begin{matrix}I_{d}& \quad \eta I_{d}\\ \eta I_{d}& \quad I_{d}\end{matrix}\bigg )\bigg ), \end{aligned}$$

similarly to Eq. 18, gives conditional distribution

$$ \textbf{v}'|\textbf{v}\sim {\text {Normal}}(\eta \textbf{v},(1-\eta ^2)\sigma ^2 I_{d}), $$

so velocities have an auto-regressive structure.

Alternatively, $\textbf{v}\sim {\text {Normal}}(\textbf{0},\sigma ^2 I_d)$ implies that the speed $s=||\textbf{v}||$ has distribution given by

$$ s/\sigma \sim \chi _d, $$

or equivalently a generalized Rayleigh distribution (Blumenson and Miller 1963), and auto-correlated speeds can be defined in terms of a suitable bivariate chi distribution. For general *d*, a suitable bivariate distribution is available from Blumenson and Miller ’s theorem (their p. 904) by setting $p=2, n=d$. In the case of most practical interest, $d=2$, the distribution of speeds is ${\text {Rayleigh}}(\sigma )$, and exactly the same approach can be used as with the reflected component of velocity at a bounce event in §4.2. If the speed before and after refreshment are *s* and $s'$ respectively, then a correlated bivariate Rayleigh distribution for $(s,s')$ implies

26 $$\begin{aligned} s'|s \sim {\text {Rice}}(s\eta , \sqrt{\sigma ^2(1-\eta ^2)}) \end{aligned}$$

as defined in §4.2.

For updating the bearing or direction of movement $\theta$, essentially any symmetric, continuous directional distribution on the $d\!-\!1$-dimensional sphere, centred on the current direction, will maintain detailed balance with respect to a uniform distribution for direction. If $d=2$, this simply means a circular distribution, and it is convenient to follow the most common practice in discrete-time ‘step and turn’ movement modelling and take the new bearing $\theta '$ to have a von Mises distribution (e.g. Evans et al. 2000, Chapter 41) with concentration parameter $\kappa$, centred on the current bearing $\theta$; that is,

$$ \theta ' \sim {\text {von Mises}}(\theta ,\kappa ), $$

or

$$ p(\theta '|\theta ) = \frac{\exp (\kappa \cos (\theta '-\theta ))}{2\pi I_0(\kappa )} $$

where $I_0(\cdot )$ is a Bessel function as in §4.2. This is the approach used in the examples in §4.5. Sampling velocity independently, as in the BPS, would then mean sampling the bearing uniformly, i.e. taking the concentration of the von Mises distribution to be zero.

### Examples of velocity-jump processes

The extended processes defined above, interpreted biologically, give a rich class of velocity-jump models in which changes in velocity can occur at completely random times, with autocorrelation in the velocities before and after the change, and can also occur in response to covariates in such a way as to respect a given utilisation distribution, with the velocity after such a change being completely general.

**Fig. 3:**
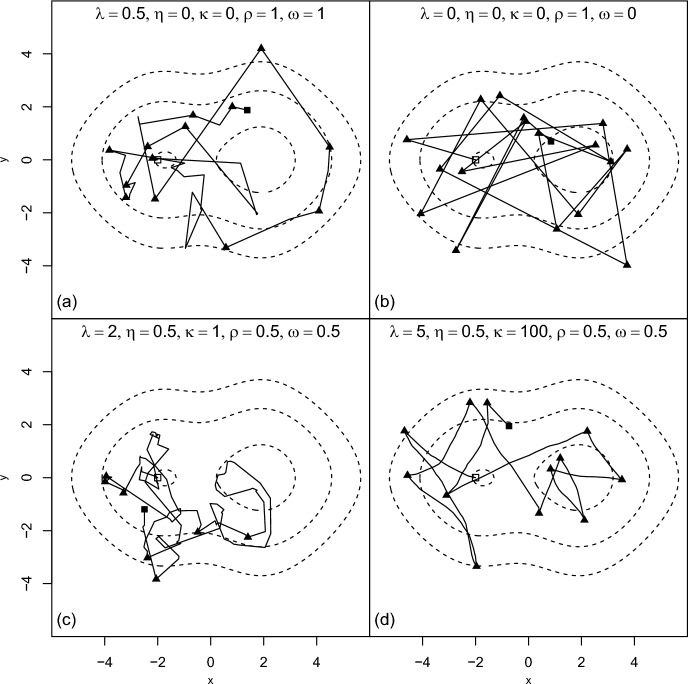
Simulations from half-space bouncy particle samplers in a 2-dimensional environment with a bimodal utilisation distribution. The parameters vary as shown; all other properties are the same for each sub-figure. Reflection events are shown by solid triangles; the dashed lines are contours of the utilisation distribution. (a) A bouncy particle sampler. (b) A generalized bouncy particle sampler. (c) A half-space bouncy particle sampler. (d) A half-space bouncy particle sampler with a higher rate of ‘refreshment’ events and a much higher concentration parameter for the turns at such events. See main text and Appendix C.3 for further details

Figure 3 shows short simulated trajectories from some example cases, to illustrate the variety of targetted models available within this class of processes. All are particular cases of the half-space bouncy particle sampler in a 2-dimensional space; where refreshment events are present, they involve conditional speeds following a Rice distribution as given in Eq. (26) and changes of bearing following a von Mises distribution with concentration $\kappa$. In each case the target is a simple bimodal distribution, for the purposes of illustration; in the target distribution, *x* and *y* co-ordinates are independent, with the *y* co-ordinate following a Logistic(0, 1) distribution and the *x* co-ordinate following a mixture of two logistic distributions, both with scale 1, with location parameters $\pm 1$ and weights 0.6, 0.4 respectively. The simulations are carried out using a uniformisation/thinning approach (Jensen 1953; Blackwell et al. 2016; Fearnhead et al. 2018); see Appendix C.3 for details.

Sub-figures (a) and (b) show examples of the BPS and GBPS respectively. The BPS has deterministic reflections, and completely random refreshments; the GBPS has some stochasticity in reflections, and no refreshments. While either could be used as a movement model, they represent quite special cases in behavioural terms. Sub-figures (c) and (d) show more general cases of the half-space bouncy particle sampler, with different parameter sets as shown. They have correlations in speed before and after refreshment events, and in both components of velocity before and after reflection events. The high refreshment rate in Fig. 3(d) leads to paths which resemble those of velocity-based diffusions, in between bounce events; as in the polar diffusion models of §3.2.3, there is a link between observed sinuosity and the rate and parameters of the refreshment process.

## Multiple behaviours

It is common for animals to exhibit variation over time in their movement patterns. Even if movement on relatively short timescales is well described by a model such as those in Sects. 2 to 4, often there is additional heterogeneity and autocorrelation over somewhat longer times; often this can be interpreted as arising from distinct behaviours by the animal at different times (e.g.  Hooten et al. 2017, §§1.1.2,5.1.5). Incorporating a discrete set of ‘behavioural’ states is a well-established means of capturing different biologically meaningful behaviours as well as other aspects of temporal auto-correlation in movement models. Since its introduction in diffusion modelling (Blackwell 1997, 2003) and discrete-time movement modelling (Morales et al. 2004), the use of multiple behavioural states has become a standard element in many approaches to movement modelling (e.g. Hooten et al. 2017; Patterson et al. 2017), including the widespread use of hidden Markov models (Morales et al. 2004). Common intuitive interpretations of the states include ‘exploratory’ and ‘encamped’ behaviours (Morales et al. 2004) or ‘resting’, ‘feeding’ and ‘running’ (Blackwell 1997). More recent developments in telemetry may allow such behavioural states to be inferred from, for example, accelerometry data, and validated against direct observation (reviewed by Brown et al. 2013).

It is straightforward to extend any of the ‘targeted’ movement models described above to have multiple states that share the same utilisation distribution. See for example §2.4.2 of Michelot et al. (2020). The states may differ in their velocity distributions, diffusion rates, etc., as appropriate, and may have quite distinct patterns of movement provided the target distribution is in common. This is analogous to the established MCMC strategy of having various types of update within the same overall algorithm, each of which maintains detailed balance with respect to the same target distribution.

More generally, models with simple movement processes and known utilisation distributions can be combined, using the concept of discrete states, to construct more complex models (Johnson et al. 2008b; Harris and Blackwell 2013; McClintock and Lander 2024); however, if the rates of switching between states do not take into account selection, then the short-term selection and the within-state and overall utilisation distributions in these models do not generally have tractable parametric forms or relate directly to the parameters or utilisation distributions of the component models. Having behavioural (or other) states with different selection parameters, while maintaining the relationship between the selection parameters and the utilisation distribution, is not completely straightforward, since the overall utilisation distribution and the within-state utilisation distributions depend on the process of switching between behaviours as well as the movement processes within behaviours. For example, Blackwell (1997) discusses cases with two states, each with movement properties derived from its own unimodal utilisation distribution, where the overall utilisation distribution may be either unimodal or bimodal, depending only on the rates of switching between states; see §2.2 and §2.5 of Blackwell (1997), building on the static models in Don and Rennolls (1983). However, it is possible to retain the meaning of the selection parameters by allowing the location and state to evolve jointly as a continuous-time Markov process on the product space $\mathbb {R}^{d} \times \{1,\ldots ,S\}$, where *S* is the number of behaviours; see Berman (1994) for technical details of some processes of this form, and Blackwell (2003) for examples without selection.

We can then define a joint utilisation distribution for location $\textbf{x}$ and state *s*, denoted $\pi (\textbf{x},s)$. For example, the natural extension of the linear-exponential form in Eq. (1) to the multistate case has

27 $$\begin{aligned} \pi (\textbf{x},s) \propto \exp \left( \beta _{s,0} + \sum _{k=1}^K \beta _{s,k}c_k(\textbf{x})\right) . \end{aligned}$$

To define a valid movement and behaviour process with joint utilisation distribution $\pi (\textbf{x},s)$, the overall dynamics must preserve that target distribution. The most straightforward and interpretable way is to require:

- within each state *s*, the movement process is consistent with the conditional utilisation distribution $\pi (\textbf{x}|s)$; and
- at each location $\textbf{x}$, the generator $\Lambda (\textbf{x})$ controlling the transition rates between states given location satisfies
  28 $$\begin{aligned} \pi (s|\textbf{x})\Lambda (\textbf{x}) = \textbf{0}, \end{aligned}$$
  where $\textbf{0}$ denotes a vector of zeroes.

Requirement (a) simply means that the movement when the behaviour is *not* changing—i.e. almost all times—should preserve $\pi (\textbf{x}|s)$, and so for each *s* it can be taken to be any of the processes from Sects. 3 and 4. Requirement (b) results in transition rates that are themselves functions of the selection parameters. Given a particular set of states and their selection parameters, a wide variety of forms for $\Lambda (\textbf{x})$ is possible. A full description of all forms of solution to Eq. (28) is beyond the scope of the current work. Such generators $\Lambda (\textbf{x})$ can be related to doubly stochastic matrices, and hence to permutations of $\{1,\ldots ,S\}$, via Birkhoff’s theorem (Birkhoff 1946; Marcus and Minc 1964), but that does not directly give a bijective parameterisation; the dimension of the space of permutations grows very rapidly compared with the space of solutions.

Many forms for $\Lambda (\textbf{x})$ can be obtained under the assumption of reversibility, so that direct transitions between states *i* and *j* satisfy ‘detailed balance’:

29 $$\begin{aligned} \pi (\textbf{x},i) \lambda _{ij}(\textbf{x}) = \pi (\textbf{x},j) \lambda _{ji}(\textbf{x}). \end{aligned}$$

Detailed balance for all pairs $i\ne j$ is a sufficient, but not necessary, condition for Eq. (28). The simplest case is to take

30 $$\begin{aligned} \lambda _{ij}(\textbf{x})&= \psi _{ij}(\textbf{x}) \sqrt{ \pi (\textbf{x},j)/\pi (\textbf{x},i)} \end{aligned}$$

where for $i\ne j$, $\psi _{ij}(\textbf{x}) =\psi _{ji}(\textbf{x})$ is a function defining the rate at which transitions occur between states *i* and *j*, in both directions, at location $\textbf{x}$, if $\pi (\textbf{x},i) = \pi (\textbf{x},j)$. Note that if $\psi _{ij}(\textbf{x})$ is written in a similar linear-exponential form to $\pi (\textbf{x},s)$ in Eq. (27), depending on spatial covariates through a set of parameters $\alpha _{ijk}$ say, then the actual rates $\lambda _{ij}(\textbf{x})$ will also have the same form, but will include the $\alpha _{ijk}, \beta _{ik}$ and $\beta _{jk}$ parameters:

31 $$\begin{aligned} \lambda _{ij}(\textbf{x}) = \exp \left( \alpha _{ij0} + \sum _{k=1}^K \alpha _{ijk}c_k(\textbf{x}) + \frac{1}{2}\left( \beta _{j0}-\beta _{i0} + \sum _{k=1}^K (\beta _{jk}-\beta _{ik})c_k(\textbf{x}) \right) \right) , \end{aligned}$$

with $\alpha _{ijk}=\alpha _{jik}$ throughout. In practice, it is likely that often simple forms for $\psi _{ij}(\textbf{x})$ would suffice, for example constant across locations and perhaps even across states. For example, if the spatial covariate effects have no effect on transition rates beyond what is necessary for consistency, then the $\alpha _{ijk}$ terms in Eq. (31) will be zero for $k=1,\ldots ,K$.

For other choices of transition rates satisfying the detailed balance defined in Eq. (29), it is helpful to exploit the analogy with MCMC methods more explicitly. Here, I am interested only in transitions between states, that is in a Markov chain on $\{1,\ldots ,S\}$. Taking inspiration from Gibbs sampling, a nominal overall rate $\kappa (\textbf{x})$ implies $\lambda _{ij}(\textbf{x}) = \kappa (\textbf{x})\pi (\textbf{x},j)/\sum _{l=1}^S\pi (\textbf{x},l)$. Alternatively, nominal rates $Q(\textbf{x}) = (q_{ij}(\textbf{x}))$ can be interpreted as proposal rates in a Metropolis-Hastings type MCMC algorithm. The usual ‘canonical’ acceptance rule for MCMC then gives

32 $$\begin{aligned} \lambda _{ij}(\textbf{x})&= q_{ij}(\textbf{x}) \min \left\{ 1,\frac{\pi (\textbf{x},j)q_{ji}(\textbf{x})}{\pi (\textbf{x},i)q_{ij}(\textbf{x})}\right\} \end{aligned}$$

where the ‘$\min$’ term corresponds to an MCMC acceptance probability. A final variation in the detailed balance case is to use non-canonical acceptance rates, which are valid for MCMC but not optimal in that context:

33 $$\begin{aligned} \lambda _{ij}(\textbf{x})&= q_{ij}(\textbf{x}) \frac{\pi (\textbf{x},j)q_{ji}(\textbf{x})}{\pi (\textbf{x},i)q_{ij}(\textbf{x})+\pi (\textbf{x},j)q_{ji}(\textbf{x})} \end{aligned}$$

say. All of these schemes have transition rates that are bounded by given nominal or proposal rates; in some cases, it may be advantageous to be able to model this bound directly, rather than using the formulation in Eq. (30). The canonical Metropolis-Hastings version will be more familiar in an MCMC context, but in comparison the non-canonical rates are smooth functions of $\textbf{x}$, provided that $\pi$ and $q_{ij}$ are smooth.

In practice many movement models involve very small numbers of states, so particular cases are of value. If $S=2$, Eq. (28) implies detailed balance. If $S=3$, there is a simple parameterisation of the whole space of valid generators, without any limitation due to detailed balance. Given any values for $\lambda _{12}(\textbf{x}), \lambda _{23}(\textbf{x}), \lambda _{31}(\textbf{x})$, and assuming $\pi (1|\textbf{x}),\pi (2|\textbf{x}),\pi (3|\textbf{x})>0$, the remaining off-diagonal elements of $\Lambda (\textbf{x})$ can be written as

$$\begin{aligned} \lambda _{21}(\textbf{x})&= \frac{\pi (1|\textbf{x})}{\pi (2|\textbf{x})}\lambda _{12}(\textbf{x}) + \epsilon (\textbf{x})/\pi (2|\textbf{x}),\\ \lambda _{32}(\textbf{x})&= \frac{\pi (2|\textbf{x})}{\pi (3|\textbf{x})}\lambda _{12}(\textbf{x}) + \epsilon (\textbf{x})/\pi (3|\textbf{x}),\\ \lambda _{13}(\textbf{x})&= \frac{\pi (3|\textbf{x})}{\pi (1|\textbf{x})}\lambda _{12}(\textbf{x}) + \epsilon (\textbf{x})/\pi (1|\textbf{x}), \end{aligned}$$

where $\epsilon (\textbf{x})\ge -\max \{\pi (1|\textbf{x})\lambda _{12}(\textbf{x}),\pi (2|\textbf{x})\lambda _{23}(\textbf{x}),\pi (3|\textbf{x})\lambda _{31}(\textbf{x})\}$ represents the non-reversible part of the transition process at $\textbf{x}$.

**Fig. 4:**
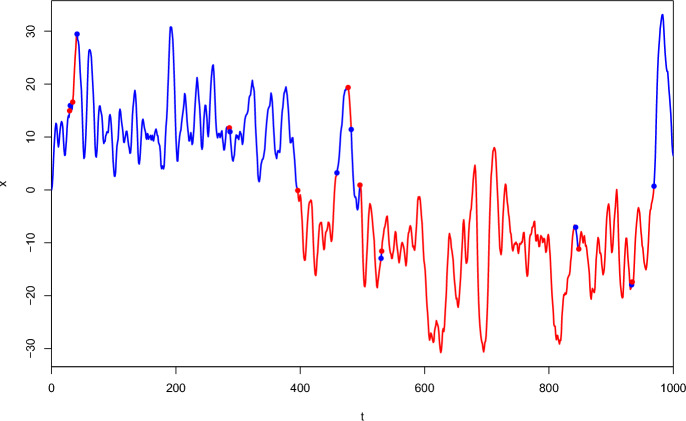
Kinetic Langevin model (Eq. (6) with two behavioural states in a 1-dimensional environment. The switching rates between states follow the ‘symmetric’ form given by Eq. (30). Behavioural state at a given time is indicated by colour; solid dots indicate transitions into the state of the given colour. The conditional utilisation distribution within each state is unimodal, with modes at -10 and 10 for states 1 (red) and 2 (blue) respectively; see text for details. For computational information, see Appendix C.4.

As an example, Fig. 4 shows a simulated trajectory in 1 dimension with $S=2$ states and with $K=2, \beta _{10}=\log (5/3), \beta _{20}=0, \beta _{11}=\beta _{22}=2, \beta _{12}=\beta _{21}=0$. Thus in state 1 the utilisation distribution is determined purely by covariate 1, and in state 2 by covariate 2. The covariates have simple forms based on Laplace distributions:

$$\begin{aligned} c_k(\textbf{x}) = \tau \exp \left( -\frac{1}{2\tau }|\textbf{x}-\mu _k|\right) \end{aligned}$$

where $\tau =10, \mu _1=-10, \mu _2=10$. The dynamics are given by the kinetic Langevin process defined in Eq. (6), with $\alpha =1, \gamma =5$ and with the gradient term $\nabla U$ based on the conditional utilisation distribution corresponding to the current state. Switching between states is given by Eq. (30) with $\psi _{12}(\textbf{x})=\psi _{21}(\textbf{x})=\sqrt{0.0015}$ for all $\textbf{x}$. The differences in distribution by behaviour can be clearly seen, and the formulation ensures that (in the long run) they exactly match what is implied by the covariates and the selection parameters within each state. Mostly the process is in state 1 when $\textbf{x}<0$ and state 2 when $\textbf{x}>0$, but visits to the opposite state, mostly short, produce the appropriate tail behaviour for the utilisation distribution within each state.

## Multiple animals

The models presented so far apply naturally to individual animals, e.g. with their own territories, or to colonies of animals that share a common utilisation distribution and move independently within it. It is however possible to extend explicit continuous-time movement models to multiple animals that interact in their movements. For example, Niu et al. (2016) modelled the interactions of *n* simultaneously-tracked reindeer in 2-dimensional space by representing them collectively as a single point following a diffusion process in a 2*n*-dimensional space. Niu et al. (2022) and Milner et al. (2021) extended this work to incorporate spatial heterogeneity and behavioural switching. None of this work included habitat selection as such, however.

To combine interaction and selection, the simplest approach would be to define a joint utilisation distribution for all *n* individuals, and add terms to the joint potential so that

34 $$\begin{aligned} \pi (\textbf{x}_1,\ldots ,\textbf{x}_n) \propto \exp \left( \sum _{i=1}^n {L}(\textbf{x}_i) + \beta _{{\text {Int}}}\sum _{i<j} \zeta (\textbf{x}_i,\textbf{x}_j)\right) , \end{aligned}$$

say, where $\textbf{x}_i$ represents the location of the *i*th individual, $\zeta (\cdot )$ is symmetric in its arguments and does not depend on the individual selection parameters, and $\beta _{{\text {Int}}}$ controls the strength of interaction. However, that approach leads to a different marginal distribution i.e. $\pi (\textbf{x})$ is no longer proportional to $\exp ({L}(\textbf{x}))$. To maintain the marginal distribution and hence the interpretation of the selection coefficients needs a more careful formulation.

One possible way is motivated by the local Gibbs sampler of §2. Reinterpreting the symmetric kernel $\psi (\cdot |\cdot )$ defined there as describing local interaction rather than movement, in the simplest case of $n=2$ we can write

35 $$\begin{aligned} \pi (\textbf{x},\textbf{y}) = \pi (\textbf{x}) \pi (\textbf{y}) \int _{\textbf{z}} \frac{\psi (\textbf{x}|\textbf{z})\psi (\textbf{y}|\textbf{z})}{\Psi (\textbf{z})}{{\text {d}}\textbf{z}} \end{aligned}$$

where

36 $$\begin{aligned} {\Psi (\textbf{z})} = \int _\textbf{u}\pi (\textbf{u}) \psi (\textbf{u}|\textbf{z}) {{\text {d}}\textbf{u}}. \end{aligned}$$

Integrating the joint distribution (35) then gives the correct marginal distribution $\pi (\textbf{x})$ for $\textbf{x}$ (and by symmetry for $\textbf{y}$). This implies that the selection parameters $\beta _k$ enter the interaction term indirectly, via $\Psi (\cdot )$. Unfortunately the joint distribution cannot be written in the convenient linear-exponential form of Eq. (34), but the strength of interaction can be controlled by allowing $\psi (\cdot |\cdot )$ to depend on a scale parameter. The model can be generalized to $n>2$, while still assuming only pairwise interaction, by writing

37 $$\begin{aligned} \pi (\textbf{x}_1,\ldots ,\textbf{x}_n) = \prod _i \pi (\textbf{x}_i) \cdot \int _\textbf{z}\left\{ \prod _{i<j} \frac{\psi (\textbf{x}_i|\textbf{z})\psi (\textbf{x}_j|\textbf{z})}{\Phi (\textbf{z})}\right\} {{\text {d}}\textbf{z}}\end{aligned}$$

where

38 $$\begin{aligned} \Phi (\textbf{z})&= m_{n-1}\Psi _{n-1}(\textbf{z})^{n-1}, \end{aligned}$$

39 $$\begin{aligned} \Psi _{n-1}(\textbf{z})&= \int _\textbf{u}\pi (\textbf{u})\psi (\textbf{u}|\textbf{z})^{n-1}{{\text {d}}\textbf{u}}, \end{aligned}$$

40 $$\begin{aligned} m_{n-1}&= \int _\textbf{u}{\psi (\textbf{u}|\textbf{0})^{n-1}} {{\text {d}}\textbf{u}}. \end{aligned}$$

The constant $m_{n-1}$ does not depend on location because of the spatial homogeneity of $\psi$. Again, this preserves the marginal distributions. Alternatively, with $n>2$ the model could be formulated in terms of interactions with an actual leader or conceptual ‘group centre’, as in Langrock et al. (2014) (in discrete time) and Niu et al. (2016). In all cases, once the joint distribution is defined in this way then $\nabla \log (\pi (\textbf{x}_1,\ldots ,\textbf{x}_n))$ can be calculated and used in any of the continuous-time movement models in Sects. 3, 4 and 5.

**Fig. 5:**
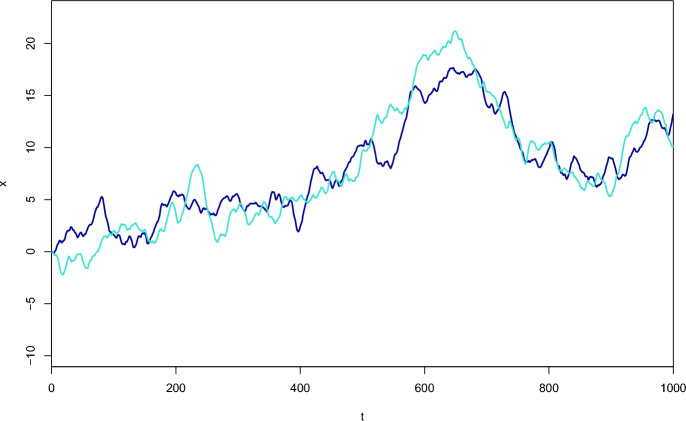
Simultaneous movement of two interacting individuals in a 1-dimensional environment, each with a bimodal utilisation distribution defined in Eq. (42). Their joint utilisation distribution is defined by Eq. (35), with the interaction kernel $\psi$ having a Gaussian shape. The dynamics within the joint utilisation distribution are defined by the kinetic Langevin process in Eq. (6). For computational details see Appendix C.5

As a simple illustration, I will consider an example in 1 dimension, with $n=2$ individuals and a single covariate. The target distribution is given by

41 $$\begin{aligned} \pi (\textbf{x})&\propto \exp (\beta _1c_1(\textbf{x})) \end{aligned}$$

42 $$\begin{aligned} c_1(\textbf{x})&= \exp \left( -\frac{1}{2\tau }\min \{|\textbf{x}-\mu _A|,|\textbf{x}-\mu _B|\}\right) \end{aligned}$$

with $\beta _1=2, \tau =10,\mu _A=-10,\mu =10$, so is bimodal with peaks at $\mu _A$ and $\mu _B$. The interaction is defined by $\psi (\textbf{y}|\textbf{x}) \propto \exp (-(\textbf{x}-\textbf{y})^2/(2{\varsigma }^2)$, with ${\varsigma }=2$. The dynamics are given by the kinetic Langevin model of Eq. (6), with $\alpha =0.01, \gamma =0.05$. Figure 5 shows a realisation of this model over a relatively short time; both the kinetic Langevin dynamics, which are smoother than the first-order Langevin case, and the correlation between the locations of the two individuals are evident.

**Fig. 6:**
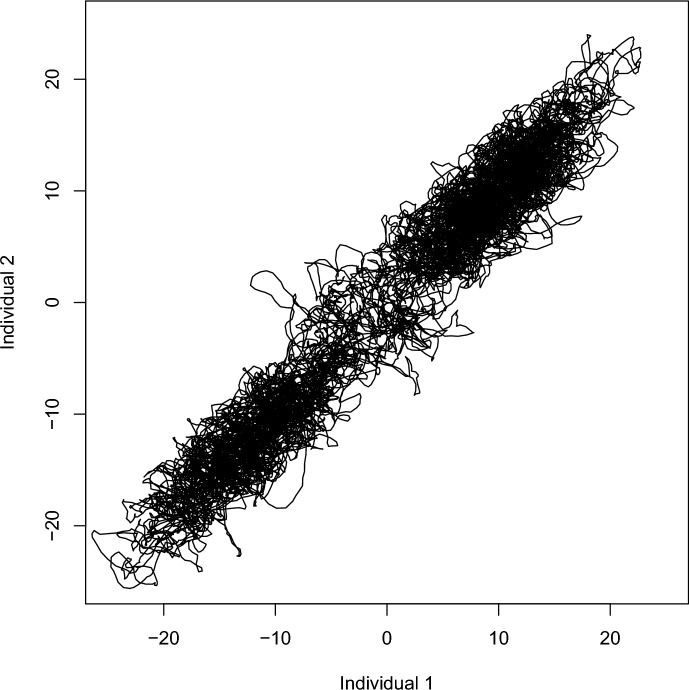
Joint locations of two interacting individuals, following kinetic Langevin dynamics within the joint utilisation distribution defined by Eq. (35), as in Fig. 5. The locations of the individuals are plotted as *x* and *y* coordinates, over a much longer simulation than in Fig. 5. The bimodality of each marginal utilisation distribution, defined in Eq. (42), is apparent, as is the correlation between indivuduals imposed by the interaction kernel $\psi$. For computational details see Appendix C.5.

Figure 6 shows the locations of the two individuals plotted against each other, for a longer run (from $t=0$ to $t= 50,000$). Again, the strong correlation between their locations is clear, as is the bimodality in the marginal locations due to the spatial selection terms derived from Eq. (42).

An additional toy example where the utilisation distribution is simple enough for all calculations to be done analytically is given in Appendix B, showing explicitly both the form of $\pi (\textbf{x},\textbf{y})$ and the failure of the naïve approach to preserve marginal distributions.

## Inference

A detailed account of inference for these models is outside the scope of the current paper, but a brief summary is given here.

Inference on the selection parameters of the discrete-time models of §2 is straightforward in principle, using explicit calculation of the likelihood, though computationally expensive (Michelot et al. 2019a, 2020). A much faster approach using variational inference is developed by Masolele et al. (2024).

For the Langevin model of §3.1, inference can be carried out using the obvious Euler-Maruyama discretisation, provided that the time interval between observations is not too short (Michelot et al. 2019b); each approximate step comes from a bivariate normal distribution, so can be calculated very rapidly. Bias in the estimation of the selection parameters is potentially an issue, but Blackwell and Matthiopoulos (2024) give a refinement to the calculation, evaluating the gradient at multiple points in a neighbourhood of each location observed, which is very effective in eliminating the bias. A similar approach should be effective for the other diffusion models described in §3. Blackwell and Matthiopoulos (2024) also carry out joint inference for telemetry data, based on the Langevin model, and spatial survey data, based directly on the corresponding stationary distribution, by combining the likelihoods. They explore the trade-offs in effort and precision between these two data types, and show that it depends on the spatial autocorrelation structure of the covariates.

For the velocity-jump models of §4, inference is more challenging. For versions without selection, inference has tended to involve limiting arguments or approximations (Ruiz-Suarez et al. 2020) or to assume that the entire trajectory is available, either by direct observation or through a separate reconstruction (Alharbi et al. 2026; Munden et al. 2021). Exact inference is possible (Blackwell 2025) using Reversible Jump MCMC (Green 1995), but computationally expensive; in principle the same approach can be extended to the targeted case, i.e. with selection, but in practice approximation based on time-discretisation, as introduced in Bray (2026), is necessary for datasets of any great size.

## Discussion

A fundamental difficulty with step selection is that it generally ignores the timescale of observations, so that selection coefficients, or other models of relative preference for the endpoints of steps, have no consistent meaning. Since habitat selection can be viewed as simply step selection on a very long timescale, this difficulty extends to any attempt to relate habitat selection based on independent observations, e.g. from spatial surveys, to step selection. While this problem has been recognised in principle for some time (Moorcroft and Barnett 2008), it has been largely ignored until relatively recently; starting with Michelot, Blackwell and Matthiopoulos (2019a), a few specific models that address this issue have been formulated and applied.

In this paper I have shown that in fact it is possible to formulate a much wider range of models that, by construction, have consistently defined movement processes (i.e. conditional distributions) and utilisation distributions (i.e. marginal distributions). They include both diffusion models and velocity-jump models, and can allow for discrete underlying behavioural states and interactions between individuals.

The models explicitly described here, and models that can constructed straightforwardly by combining them, can express a wide range of movement patterns. For example, the diffusion models of Eqs. (5) and (12), in which selection manifests only in the drift and diffusion terms repectively, are simply endpoints of a possible continuum of models, and the results of Ma et al. (2019) can be used to parameterise that continuum. Similarly, by using the ideas of §5, it is possible to combine very different movement processes—even diffusion and velocity-jump processes—in a single model in a coherent way.

The models described here provide the necessary framework and toolkit to greatly extend current research approaches of great practical importance: not just the analysis of individual movement data, as in many of the papers cited above, but also the combination of movement and spatial survey data (Blackwell and Matthiopoulos 2024) and the simultaneous modelling of individuals that are both interacting and exhibiting resource selection.

This general framework necessitates a more careful consideration of how covariates used in modelling movement and space use are obtained and derived (c.f. Niebuhr et al. 2023). For proper consistency and interpretation, it seems likely that in many cases, derived covariates, particularly based on spatial smoothing, will be more appropriate than ‘raw’ covariates measured directly. This follows from modelling the locations where animals spend their time, as opposed to simply the locations that are of direct interest to them; it leads to covariates that depend on the spatial pattern of physical and biological features, but that reflects the necessity of animals travelling through space rather than simply appearing at locations of direct interest to them.

Movement models are essentially about conditional distributions of the form $p(\textbf{x}(t+\delta t)|\textbf{x}(t))$, and, as mentioned in Sect.1, it is usually appropriate to think of $\delta t$ as arbitrary; it generally has no biological meaning, and often arises from the pattern of observation times. One obvious exception is where interest really is focussed only on locations at regular time intervals, for example in studying the distribution of locations where (diurnal) animals spend the night, and where an observation at, say, midnight can be taken as indicative of that night’s locations. Then a discrete-time model is wholly appropriate; even then, care is needed in relating conditional distributions to asymptotic behaviour, as shown in §2.

It is also possible for the movement process to have some biologically meaningful pattern on a relatively fine timescale, for example due to an individual’s actual steps, hops or even wingbeats. Such a pattern is unlikely to be exactly regular, but $p(\textbf{x}(t+\delta t)|\textbf{x}(t))$ may depend strongly on how $\delta t$ relates to the timescale of the pattern. Often in such cases, it will be sensible to neglect that structure, essentially modelling a version of the movement that is smoothed over that fine timescale; if not, it may be necessary to augment the underlying state space not only with velocity, as in various models presented above, but also with the individual’s phase within the process defined by its gait.

More generally, time-varying patterns of selection may be hard to formalise in a way that is consistent when considered at different timescales. When selection involves time-varying covariates, but the utilisation distribution is not of interest (or not well-defined), it may be sufficient to formulate a model in continuous time to avoid contradictions between different timescales. Otherwise, for very specific cases, some partial results, both exact and approximate (not detailed here) are available, but extending the wider framework presents some challenging open questions.

## Figures and Tables

**Fig. 7:**
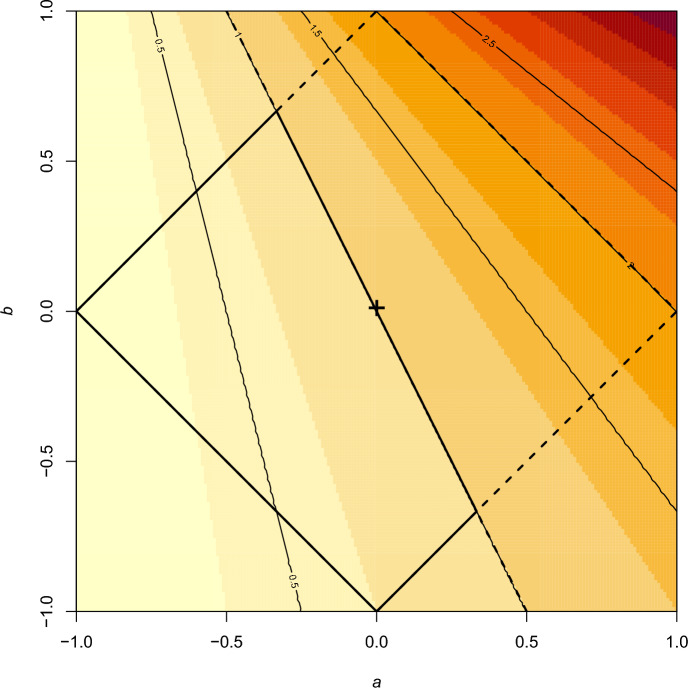
The feasible region for reorientation updates. The heatmap and thin solid contour lines show *h* as a function of *a* and *b*. The dashed lines show the constraints on $|a|+|b|$ and on |*h*|, and the thicker solid lines show the region where both constraints are satisfied. The vertices of the latter region and the origin (marked by $\mathbf {+}$) are described in Table 1 in §4.3

**Algorithm 1:**
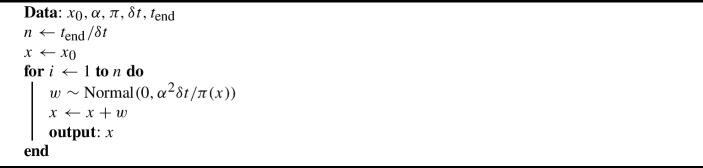
Simulation of 1-d diffusion process with varying diffusion rate

**Algorithm 2:**
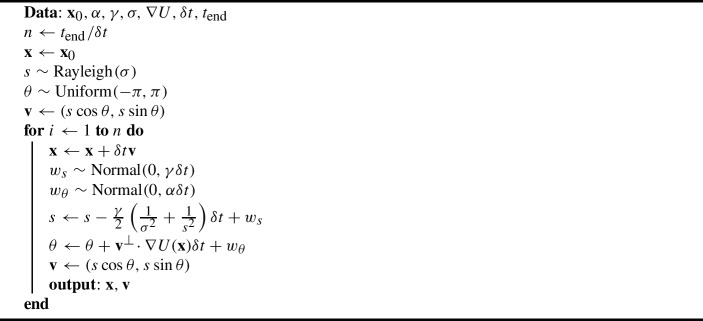
Simulation of polar velocity diffusion process

**Algorithm 3:**
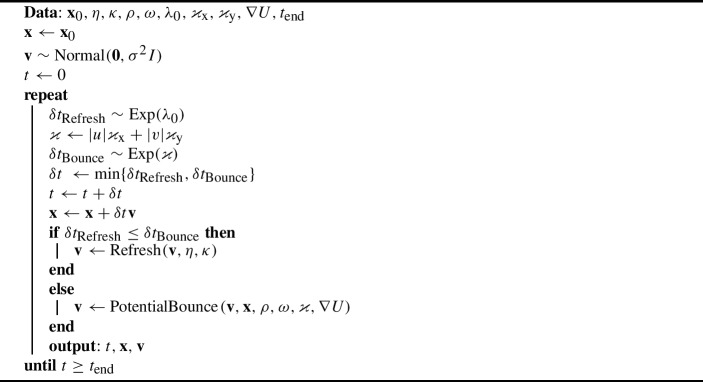
Simulation of bouncy particle sample and extensions

**Algorithm 4:**
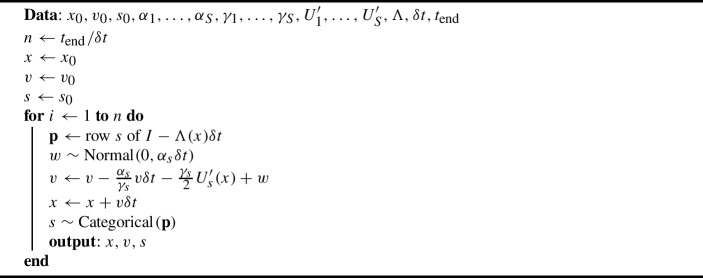
Simulation of a 1-d kinetic Langevin model with *S* states

**Algorithm 5:**
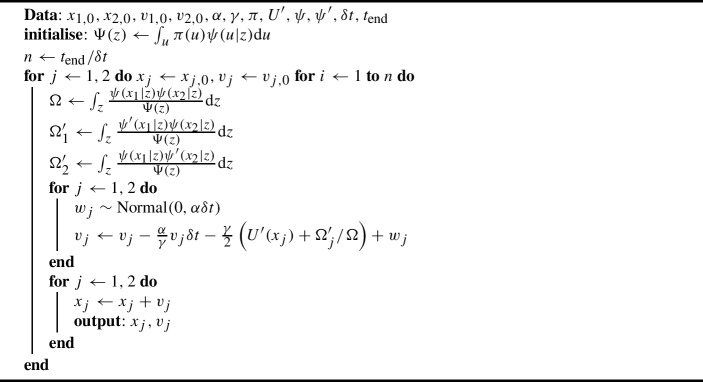
Simulation of two interacting 1-d diffusion processes

## Data Availability

No data were used in this research.

## Reorientation models with full support

From §4.3 of the main text, the valid relationships between the coefficients in Eq. (22) for the rate of reorientations and Eq. (23) for the distribution of bearings are given by Eq. (24), subject to the conditions on their absolute values. Rearranging Eq. (24) gives $h = (1+a)/(1-b/2),$ and Figure 7 shows values of *h* as a function of *a* and *b*.

## Analytic example of interaction

Let $\pi (\textbf{x}) = \pi (\textbf{y}) = {\text {N}}(0,\tau ^2), \psi (\textbf{y}|\textbf{x}) = {\text {N}}(\textbf{x},\sigma ^2)$ in Eq. (27). Then integrating analytically gives $\Psi (\textbf{z}) = {\text {N}}(0,\sigma ^2+\tau ^2)$. Explicitly calculating the joint distribution from Eq. (27) gives

$$ (\textbf{x},\textbf{y}) \sim {\text {N}}(\textbf{0},V) $$

with

$$ V = \tau ^2\begin{pmatrix}1& \rho \\ \rho & 1\end{pmatrix} $$

where the correlation is given by $\rho =\tau ^2/(\sigma ^2+\tau ^2)$. The distribution of $\textbf{x}-\textbf{y}$ is ${\text {N}}(0,2\sigma ^2\rho )$.

For comparison, the naïve case based on Eq. (34) with

43 $$\begin{aligned} \pi (\textbf{x},\textbf{y}) \propto \exp \left( -\frac{1}{2}\left\{ \frac{\textbf{x}^2}{\tau ^2}+\frac{\textbf{y}^2}{\tau ^2}+\frac{(\textbf{x}-\textbf{y})^2}{\sigma ^2}\right\} \right) \end{aligned}$$

gives

44 $$\begin{aligned} (\textbf{x},\textbf{y})&\sim {\text {N}}(\textbf{0},\widetilde{V}) \end{aligned}$$

45 $$\begin{aligned} \widetilde{V}&= \frac{\tau ^2(\sigma ^2+\tau ^2)}{\sigma ^2+2\tau ^2}\begin{pmatrix}1& \rho \\ \rho & 1\end{pmatrix} \end{aligned}$$

so the marginal distributions for $\textbf{x}$ and $\textbf{y}$ have variance reduced by the presence of the interaction term.

Of course, in this tractable toy example it is very simple to correct for this; the point is that in general, the formulation in Eq. (27) maintains the marginal distribution *automatically*.

## Computational details

Pseudocode is provided here for all the models used for the simulations in the figures in the main text. The actual simulations were carried out in R (R Core Team 2024); for further details please contact the author.

### Diffusion in 1 dimension with varying diffusion rate

Figure 1 shows a realisation of a 1-d diffusion with zero drift in which the diffusion rate is inversely proportional to the utilisation distribution, as defined in Eq. (12) in §3.2.1. The simulation uses standard Euler-Maruyama approximation in 1 dimension.

**Table 2:** Inputs to the simulation of 1-d diffusion process, with varying diffusion rate

**Input**	**Meaning**
$x_{0}$	Initial location of animal
$\alpha$	Parameter controlling diffusion rate
$\pi$	Function defining utilisation distribution
$\delta t$	Small time increment
$t_{\textrm{end}}$	Duration of simulation

### Polar velocity diffusion

Figure 2 shows realisations of a 2-d diffusion model where the state consists of location, speed and bearing, as defined in Eqs. (15) and (16) in §3.2.3. The simulation uses standard Euler-Maruyama approximation in 2 dimensions.

The cases shown in the figure differ in their values of $\alpha$, the diffusion rate of the bearing, and all have $\gamma =0$, so that speed is constant. The pseudocode covers the more general case where speed has a Rayleigh($\sigma$) distribution and follows a 1-d Langevin process, as described in the main text.

**Table 3:** Inputs to the simulation of a 2-d diffusion process with polar velocity

**Input**	**Meaning**
$\textbf{x}_{0}$	Initial location of animal
$\alpha$	Diffusion rate of bearing $\theta$
$\gamma$	Parameter of the 1- Langevin process for speed *s*
$\sigma$	Scale parameter of Rayleigh distribution of speed *s*
$\nabla U$	Function giving vector gradient of logarithm of utilisation distribution
$\delta t$	Small time increment
$t_{\textrm{end}}$	Duration of simulation

### Bouncy particle sampler and extensions

Fig. 3 in §4.5 shows simulations from the Bouncy Particle Sampler (BPS), the Generalized BPS, and the Half-space BPS, defined in §4. All are implemented as special cases of the Half-space BPS, as described in §4.2.

Purely for computational purposes, the algorithm here makes the assumption, valid for all the examples in the text, that the gradient of the log of the target distribution is bounded, and hence, for any given velocity, the rate of bounces is also bounded as a function of location. That means that bounce events can be simulated using a uniformisation technique. If the velocity is $\textbf{v}$, and $\lambda (\textbf{x},\textbf{v})\le \varkappa (\textbf{v})$ for all $\textbf{x}$, then events at a rate $\lambda (\textbf{x},\textbf{v})$ can be generating by simulating a Poisson process of rate $\varkappa (\textbf{v})$, regardless of changes in $\textbf{x}$ over time, and then thinning that process, retaining an event at location $\textbf{x}$ with probability $\lambda (\textbf{x},\textbf{v})/\varkappa (\textbf{v})$ (Jensen 1953; Blackwell et al. 2016; Fearnhead et al. 2018). In the absence of such bounds, the models are still valid, but simulation from them involves additional complexity.

In the PotentialBounce function, the notation $\textbf{v}^\perp$ represents a vector perpendicular to $\textbf{v}$ and of the same length. The choice between the two possible directions is arbitrary, provided it is consistent.

**Table 4:** Inputs to the simulation of the half-space bouncy particle sampler

**Input**	**Meaning**
$\textbf{x}_{0}$	Initial location of animal
$\kappa ,\lambda _0$	Parameters of the ‘refreshment’ process
$\eta ,\rho ,\omega$	Parameters of the half-space ‘bounce’ process
$\varkappa _\textrm{x}, \varkappa _\textrm{y}$	Upper bounds on the absolute value of each component of $\nabla U$
$\nabla U$	Function giving vector gradient of logarithm of utilisation distribution
$t_{\textrm{end}}$	Duration of simulation

### Multiple behaviours

Figure 4 shows a simulation of an individual moving around a 1-dimensional utilisation distribution while switching between two different behavioural states, as described in Sect. 5. Within each state, the animal follows the kinetic Langevin process defined in Eq. (6); switching between states is defined by Eq. (30).

**Table 5:** Inputs to 1-d simulation with multiple behaviours

**Input**	**Meaning**
$x_0$	Initial location
$v_0$	Initial speed
$s_0$	Initial behavioural state
$\alpha _s, \gamma _s, s=1,\ldots ,S$	Parameters of kinetic Langevin movement in state *s*
$U'_s, s=1,\ldots ,S$	Function defining derivative of logarithm of conditional utilisation distribution in state *s*
$\Lambda$	Function defining the transition rate matrix between states as a function of *x*
$\delta t$	Small time increment
$t_{\textrm{end}}$	Duration of simulation

### Interacting animals

Figures 5 and 6 show simulations of different duration from the same model, in which two individuals move around a 1-d utilisation distribution while also attracted towards each other, as defined in Eqs. (35) and (36) in Sect. 6. The dynamics are given by the kinetic Langevin process defined in Eq. (6). The simulation uses standard Euler-Maruyama approximation, but the calculation of the drift term from the gradient of the joint utilisation distribution is quite complex.

Numerical integration was used for all the integrals in this algorithm. The ‘initialisation’ of $\Psi$ involved integrations at a fine grid of *z* values, defining a function of *z* stored as an array for linear interpolation; in R, this used the function approxfn. All numerical integration in the example used integrate in R, with default settings. The gradient $U'$ was calculated numerically; for the example in the text, $\psi$ has a Gaussian form and so the derivative $\psi '$ is available in closed form.

**Table 6:** Inputs to the simulation of interacting animals

**Input**	**Meaning**
$x_{1,0},x_{2,0}$	Initial locations of animals
$v_{1,0},v_{2,0}$	Initial speeds of animals
$\alpha ,\gamma$	Parameters of individual kinetic Langevin model
$\pi$	Function defining marginal utilisation distribution
$U'$	Function defining derivative of logarithm of $\pi (\cdot )$
$\psi$	Function defining interaction term
$\psi '$	Function defining first partial derivative of $\psi$
$\delta t$	Small time increment
$t_{\textrm{end}}$	Duration of simulation
